# Phase I study evaluating the Fc-optimized FLT3 antibody FLYSYN in AML patients with measurable residual disease

**DOI:** 10.1186/s13045-023-01490-w

**Published:** 2023-08-17

**Authors:** Jonas S. Heitmann, Richard F. Schlenk, Daniela Dörfel, Sabine Kayser, Konstanze Döhner, Michael Heuser, Felicitas Thol, Silke Kapp-Schwoerer, Jannik Labrenz, Dominic Edelmann, Melanie Märklin, Wichard Vogel, Wolfgang Bethge, Juliane S. Walz, Ludger Große-Hovest, Martin Steiner, Gundram Jung, Helmut R. Salih

**Affiliations:** 1 Clinical Collaboration Unit Translational Immunology, German Cancer Consortium (DKTK), Department of Internal Medicine, University Hospital Tübingen, University of Tübingen, Tübingen, Germany; 2 Cluster of Excellence iFIT (EXC2180) “Image-Guided and Functionally Instructed Tumor Therapies”, University of Tübingen, Tübingen, Germany; 3 NCT Trial Center, National Center for Tumor Diseases, German Cancer Research Center (DKFZ) and Heidelberg University Hospital, Heidelberg, Germany; 4 Department of Internal Medicine V, Heidelberg University Hospital, Heidelberg, Germany; 5 Department of Hematology, Oncology and Immunology, KRH Klinikum Siloah, Hannover, Germany; 6 Medical Clinic and Policlinic 1, Hematology, Cellular Therapy and Hemostaseology, University of Leipzig Medical Center, Leipzig, Germany; 7 Institute of Transfusion Medicine and Immunology, Medical Faculty Mannheim, Heidelberg University, German Red Cross Blood Service Baden-Württemberg-Hessen, Mannheim, Germany; 8 Department of Internal Medicine III, University Hospital of Ulm, Ulm, Germany; 9 Department of Hematology, Hemostasis, Oncology, and Stem Cell Transplantation, Hannover Medical School, Hannover, Germany; 10 Division of Biostatistics, German Cancer Research Center (DKFZ), Heidelberg, Germany; 11 Department of Hematology, Oncology, Clinical Immunology and Rheumatology, University Hospital Tübingen, Tübingen, Germany; 12 Department of Peptide-Based Immunotherapy, University and University Hospital Tübingen, Tübingen, Germany; 13 Institute for Cell Biology, Department of Immunology, University of Tübingen, Tübingen, Germany; 14 German Cancer Consortium (DKTK) and German Cancer Research Center (DKFZ), Partner Site Tübingen, Tübingen, Germany; 15 Synimmune GmbH, Tübingen, Germany

**Keywords:** AML, MRD, FLT3, Fc-optimized antibody, Immunotherapy

## Abstract

**Background:**

About half of AML patients achieving complete remission (CR) display measurable residual disease (MRD) and eventually relapse. FLYSYN is an Fc-optimized antibody for eradication of MRD directed to FLT3/CD135, which is abundantly expressed on AML cells.

**Methods:**

This first-in-human, open-label, single-arm, multicenter trial included AML patients in CR with persisting or increasing MRD and evaluated safety/tolerability, pharmacokinetics and preliminary efficacy of FLYSYN at different dose levels administered intravenously (cohort 1–5: single dose of 0.5 mg/m^2^, 1.5 mg/m^2^, 5 mg/m^2^, 15 mg/m^2^, 45 mg/m^2^; cohort 6: 15 mg/m^2^ on day 1, 15 and 29). Three patients were treated per cohort except for cohorts 4 and 6, which were expanded to nine and ten patients, respectively. Primary objective was safety, and secondary efficacy objective was ≥ 1 log MRD reduction or negativity in bone marrow.

**Results:**

Overall, 31 patients were treated, of whom seven patients (22.6%) experienced a transient decrease in neutrophil count (two grade 3, others ≤ grade 2). No infusion-related reaction or dose-limiting toxicity was observed. Adverse events (AEs) were mostly mild to moderate, with the most frequent AEs being hematologic events and laboratory abnormalities. Response per predefined criteria was documented in 35% of patients, and two patients maintained MRD negativity until end of study. Application of 45 mg/m^2^ FLYSYN as single or cumulative dose achieved objective responses in 46% of patients, whereas 28% responded at lower doses.

**Conclusions:**

FLYSYN monotherapy is safe and well-tolerated in AML patients with MRD. Early efficacy data are promising and warrant further evaluation in an up-coming phase II trial.

*Trial registration* This clinical is registered on clinicaltrials.gov (NCT02789254).

**Supplementary Information:**

The online version contains supplementary material available at 10.1186/s13045-023-01490-w.

## Background

AML is primarily a disease of adults (median age at diagnosis 68 years) with a high unmet medical need [1]. Treatment with curative intention comprises intensive induction chemotherapy to achieve morphological complete remission (CR), followed by consolidation chemotherapy, allogeneic hematopoietic-cell transplantation (allo-HCT), or both [2]. Patients not eligible for intensive induction therapy due to age and comorbidities are often treated with a combination of venetoclax and hypomethylating agents. MRD frequently (about 40%) remains detectable after achieving morphological CR and constitutes the basis for relapse [3–6]. Until recently, no treatment was established for patients with MRD, with exception of HCT that is associated with substantial morbidity and mortality [7–9]. Recently, relapse-free survival was reported to be prolonged by oral azacitidine as maintenance therapy after successful first-line therapy [10].

The introduction of monoclonal antibodies (mAbs) has largely improved treatment options and outcome of cancer patients [11]. While mAb treatment meanwhile constitutes a standard of care in lymphoid neoplasia particularly of B-cell origin (e.g., Rituximab [12]), mAb immunotherapy is so far not established in myeloid malignancies in general and in AML in particular. Monoclonal Abs directed to various targets have been evaluated preclinically and clinically, including CD33, CD123 and CD38 [13], as exemplified by results obtained with lintuzumab (anti-CD33), which failed to achieve beneficial effects in AML [14]. Notably, the antibody–drug conjugate Gemtuzumab ozogamicin (anti-CD33) is approved in AML, but anti-leukemic effects are accompanied by toxicity particularly at higher doses, leading to temporary withdrawal from market use, and its mechanism of action does not involve induction of anti-tumor immune response [15].

FMS-like tyrosine kinase 3 (FLT3, CD135) is a surface expressed member of the class III receptor tyrosine kinase family. In healthy cells, low levels of FLT3 are expressed on immature hematopoietic progenitors, dendritic cells and monocytes [16–18]. In contrast, substantial FLT3 levels are expressed on leukemic cells in almost all AML patients, and binding to FLT3 is not affected by activating mutations in the *FLT3* gene [19–22]. Based on this favorable expression pattern, a mAb targeting FLT3 (LY3012218) was developed and clinically evaluated in refractory and relapsed AML, but failed to achieve clinical efficacy [23]. Induction of antibody-dependent cellular cytotoxicity (ADCC) constitutes a major mechanism by which mAbs mediate their efficacy [24–26]. Among others, tumor burden, the ratio of malignant and immune effector cells, and the capacity of a given mAb to stimulate Fcγ receptor bearing immune effector cells determine therapeutic efficacy of ADCC. This prompted the development of mAbs with genetically modified Fc-parts to increase affinity to the Fcγ receptor CD16 and thus immunostimulatory capacity [27]. In turn, the failure of LY3012218 may be explained by the fact that it contains an unmodified/non-optimized Fc-part and was evaluated in patients with high leukemic burden [23, 28]. Here, we report on the first clinical evaluation of FLYSYN, a chimeric FLT3 mAb which contains the amino acid modifications S240D and I333E (SDIE) in the CH2 domain to improve binding to CD16 and has demonstrated highly promising preclinical efficacy [22]. The optimized capacity of FLYSYN to induce ADCC and the reasoning to treat patients with low tumor burden led us to conduct a first-in-human trial evaluating safety and preliminary efficacy in AML patients in CR with detectable MRD.

## Methods

### Patients

Eligible patients, presented with histologically confirmed AML by WHO criteria [29], were aged ≥ 18 years and had an ECOG performance status of 0–2. Morphological CR according to ELN definition [30] after any therapy except for allo-HCT with stable or increasing MRD in two sequential measurements determined by central RT-qPCR and/or NGS constituted the main inclusion criterion (either in peripheral blood (PB) or bone marrow (BM) or both) [31–33]. FLT3 expression had to be confirmed on leukemic blasts. Details on inclusion and exclusion criteria are provided in Additional file 1.

All patients provided written informed consent prior to enrolment. The study was approved by the leading ethics committee of the University Hospital Tübingen (184/2016AMG1), local ethics committees and the Paul Ehrlich Institute (2893). The trial was registered on clinicaltrials.gov (NCT02789254) and EudraCT number 2016-000236-17.

### Trial design and regimes

Results of an open-label, single-arm, first in man multicenter trial (recruitment period February 2017 to March 2020) evaluating safety/tolerability and efficacy of FLYSYN in AML patients with persistent or increasing MRD are reported.

Dose escalation followed a standard 3 + 3 design. Patients were enrolled in six dosing cohorts, with cohorts 1–5 evaluating a single FLYSYN application and up to three mAb treatments in cohort 6. FLYSYN was administered i.v. over 3 h with a fixed dose on day 1 (0.5 mg/m^2^) and the remaining dose of the respective dose level applied on day 2. In cohort 1, a single dose of FLYSYN of 0.5 mg/m^2^ body surface area (BSA) was applied, whereas patients in cohorts 2, 3, 4 and 5 received a total dose of 1.5 mg/m^2^ BSA, 5 mg/m^2^ BSA, 15 mg/m^2^ BSA, 45 mg/m^2^ BSA, respectively. In cohort 6, patients received 15 mg/m^2^ BSA every 2 weeks for a total of three applications. In the absence of dose-limiting toxicity (DLT), three patients were treated per cohort except for cohorts 4 and 6, which were expanded to 9 and 10 patients, respectively (Additional file 1: Fig. S1).

Treatment-emergent adverse events (AEs) and treatment-related AEs were summarized as per the Medical Dictionary for Regulatory Activities and the National Cancer Institute Common Terminology Criteria for AEs (CTCAE) version 4.03. Safety data are summarized by counting every respective AE (SOC and lowest level term) that occurred in a patient only once. If the same AE occurred more than once, only the highest graded AE was counted. Details on trial methods are provided in Additional file 1.

### Endpoints and assessments

Primary objective was safety of FLYSYN monotherapy at various dose levels. Primary endpoint was incidence and severity of AEs over 28 days (i.e., Visit 7, day 29) in cohorts 1–5 and over 35 days after last dosing (i.e., Visit 9a, day 64) in cohort 6. Secondary safety endpoint was incidence and severity of AEs until day 180 days (Visit 11) after first dosing. DLTs (detailed description in Additional file 1) were assessed until day 15 after first FLYSYN application in cohorts 1–5 and day 43 for cohort 6. Further, secondary objectives were preliminary activity of FLYSYN as per analysis of overall molecular response rate (per study protocol defined as any ≥ 1 log MRD reduction or negativity in BM), duration of response, and time to evidence of progressive disease (EPD). Time to EPD was defined as number of days from first study drug application to the earliest evidence of progress (MRD increase by ≥ 1 log, morphological relapse or death). In addition, MRD reduction defined as any reduction from MRD at baseline was assessed. In case of qPCR, copy numbers of mutated NPM1/10,000 ABL1 copies were determined, with the threshold to define MRD positivity being > 0 NPM1/10,000 ABL copies and sensitivity depending on ABL values (range 10^−5^ and 10^−6^). When NGS was used for detection of MRD, positivity was defined as any result above the threshold of 0.01% with sensitivity being 10^−4^. Immunogenicity was assessed as percentage of subjects who develop anti-drug antibodies (ADAs). Pharmacodynamics comprised analysis of B, T and NK cell populations and activation.

### Statistical analysis

The clinical data cut-off date was February 2022. The number of patients in each cohort was based on toxicities observed as the trial progressed. Up to 31 patients were planned for enrolment. Safety, pharmacokinetics, and anti-leukemic activity analyses were done per protocol on all patients who received at least one dose of FLYSYN. All patients with drop-outs prior to end of study visit were assessed until last scheduled visit. Demographics were analyzed by descriptive statistics. Time to EPD between groups was compared using a logrank test. 95% CIs for median survival were calculated based on a log–log transform of the survival function estimate. All statistical analyses were done with SAS 9.4.

## Results

### Patient characteristics

**Fig. 1:**
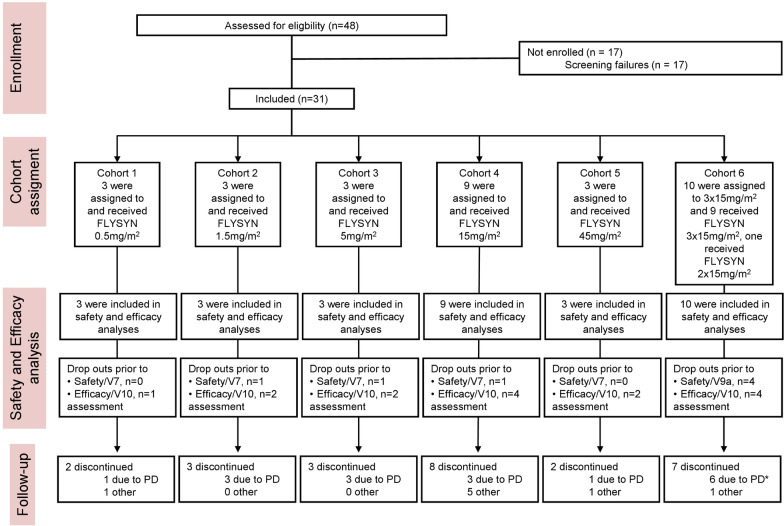
Consort flow diagram. Seventeen patients did not meet the inclusion criteria at screening and accordingly were not enrolled in the trial. Three patients underwent screening procedures twice prior to enrollment. 31 enrolled patients received FLYSYN at the indicated dose level of the assigned cohort. Safety oversight to proceed to the next higher dose cohort was performed by an independent data and safety monitoring board after an interim safety analysis of the first three study patients included in a dose level, evaluated on day 15 (cohorts 1–5) or day 43 (cohort 6) after FLYSYN application. Assessment of DLT could be done for all dose levels, whereas single patients of each cohort were not assessable at the primary safety endpoint, and 15 patients left follow-up during the assessment of efficacy for the secondary endpoint analysis. In cohort 6, one patient (asterisk) dropped out prior to third application of FLYSYN due to hematologic relapse. One patient was lost to follow-up. *n* number

From February 15, 2017, through March 18, 2020, 48 patients underwent screening and 31 were included in the intention-to-treat population at five sites in Germany (Fig. 1). All 31 patients suffered from de novo AML. The age ranged from 21 to 80 years (median 59 years) with 65% being female. A total of 55% of patients had received standard 3 + 7 induction therapy followed by consolidation therapy (for details, see Additional file 1: Table S13), and at baseline, all patients had CR with at least one detectable MRD marker. Twenty-eight, two and one participants were positive for mutated *NPM1*, mutated *IDH2* and *RUNX1-RUNX1T1*, respectively. For *NPM1*, MRD values ranged from 5 to 656,344 mutated *NPM1* copies/10,000 *ABL1* copies. *FLT3* mutations were detected in nine (29%) patients comprising *FLT3*-ITD (*n* = 5, 16% of all patients) and *FLT3*-TKD (*n* = 4, 13% of all patients). The clinical characteristics are summarized in Table 1.

**Table 1:** Patients’ characteristics

Characteristic	Value (*n* = 31)
Median age—years (range)	59 (21–80)
ECOG score—*n* (%)	
0	22 (71)
1	9 (29)
Gender—*n* (%)	
Female	20 (65)
Male	11 (35)
AML type—*n* (%)	
De novo	31 (100)
Secondary	0 (0)
Prior treatment—*n* (%)	
3 + 7^‡^	17 (55)
3 + 7 + midostaurin^‡^	3 (10)
A-ICE^‡^	6 (19)
Other*	5 (16)
Mutations—*n* (%)	
*FLT3*-ITD	5 (15)
*FLT3*-TKD	4 (13)
MRD Marker—*n* (%)	
*NPM1*	28 (90)
*IDH2*	2 (6)
*RUNX1-RUNX1T1*	1 (3)
*DTA mutations*^a^	0 (0)
MRD baseline values	
*NPM1* MRD median (range) (qPCR)^⁑^	936 (5—656,344)
IDH2 MRD median (range) (NGS)^†^	0.729 (0.2–1.258)
*RUNX1-RUNX1T1* MRD median (range) (NGS)^†^	0.06 (n.a.)

*A-ICE* all-trans retinoic acid, idarubicine, cytarabine, etoposide, *AML* acute myeloid leukemia, *ECOG* Eastern Cooperative Oncology Group, *FLT3* fms like tyrosine kinase 3, *MRD* minimal residual disease, *n* number, *n.a.* not applicable, *NGS* next generation sequencing, *qPCR* quantitative polymerase chain reaction

^*^Other prior treatments include treatment regimens with cytarabine, etoposide, all-trans retinoic acid, decitabine, gemtuzumab ozogamicin

^‡^Induction therapy was followed by high dose cytarabine consolidation

^⁑^qPCR is assessed in mutated NPM1 copies/10,000 *ABL1* copies

^†^NGS is assessed in % of mutated/variant allele frequency

^a^DTA includes mutations in DNMT3A, TET2 and ASXL1

### Safety and tolerability

No DLT was reported for FLYSYN at any dose cohort. For the primary safety endpoint, treatment-emergent AEs were observed in 25 (81%) of the 31 patients, of which in 15 (48.5%) patients AEs were considered related to FLYSYN (Table 2 and Additional file 1: Table S1). The most common any-grade treatment-emergent AEs in all cohorts were laboratory abnormalities and hematologic events [neutrophil count decreased (22.6%), anemia (19.4%) and white blood cell decreased (19.4%)], of which most were grade 1–2 (Table 2). The only grade 3 treatment-emergent AEs was neutropenia (6.5%, one in cohort 4 and cohort 6, each) and back pain (3.2%, one in cohort 6) (Additional file 1: Tables S2 and S3). Only three patients (9.7%) developed fever after infusion, but none above grade 1 (Table 2). Most common treatment-related AEs of any grade were decreased neutrophil count (22.6%), anemia (19.4%) and decreased white blood cell count (19.4%, Additional file 1: Table S1). No support with G-CSF was required, and no neutropenic fever was observed. In the 18 patients receiving FLYSYN at ≤ 15 mg/m^2^ (cohorts 1–4), most common treatment-emergent AEs were anemia (11.1%), constipation (11.1%), hypokalemia (11.1%), hypertension (11.1%), decrease in neutrophil (11.1%) and platelet (11.1%) count. Of those, anemia (11.1%), decreased neutrophil count (5.6%) and decreased platelet count (11.1%) were considered related to FLYSYN (Additional file 1: Tables S4 and S5). In the 13 patients receiving > 15 mg/m^2^ of the drug (cohorts 5 and 6), most common treatment-emergent AEs were decreased neutrophil (46.2%) and white blood cell count (46.2%), anemia (30.8%), fatigue (30.8%), decreased lymphocyte count (30.8%) and pain in extremity (30.8%). Of those, all hematologic events were assessed as related to FLYSYN (Additional file 1: Tables S4 and S5).

**Table 2:** Treatment-related adverse events for all cohorts

SOC	CTCAE term	All subjects (*n* = 31)			
		Grade 1	Grade 2	Grade 3	Any grade
Patients with events	All terms	8 (25.8)	4 (12.9)	3 (9.7)	15 (48.4)
Blood and lymphatic system disorders	Anemia, *n* (%)	5 (16.1)	1 (3.2)	0	6 (19.4)
Gastrointestinal disorders	Nausea, *n* (%)	1 (3.2)	0	0	1 (3.2)
General disorders and administration site conditions	Chills, *n* (%)	2 (6.5)	0	0	2 (6.5)
	Edema limbs, *n* (%)	1 (3.2)	0	0	1 (3.2)
	Fatigue, *n* (%)	3 (9.7)	0	0	3 (9.7)
	Fever, *n* (%)	3 (9.7)	0	0	3 (9.7)
	Flu like symptoms, *n* (%)	2 (6.5)	0	0	2 (6.5)
	Non-cardiac chest pain, *n* (%)	1 (3.2)	0	0	1 (3.2)
Investigations	Blood bilirubin increased, *n* (%)	2 (6.5)	0	0	2 (6.5)
	Investigations—Other, interleukin 2 receptor increased, *n* (%)	1 (3.2)	0	0	1 (3.2)
	Lymphocyte count decreased, *n* (%)	3 (9.7)	1 (3.2)	0	4 (12.9)
	Neutrophil count decreased, *n* (%)	2 (6.5)	3 (9.7)	2 (6.5)	7 (22.6)
	Platelet count decreased, *n* (%)	2 (6.5)	0	0	2 (6.5)
	WBC decreased, *n* (%)	3 (9.7)	3 (9.7)	0	6 (19.4)
Musculoskeletal and connective tissue disorders	Back pain, *n* (%)	0	0	1 (3.2)	1 (3.2)
	Pain in extremity, *n* (%)	1 (3.2)	1 (3.2)	0	2 (6.5)
Nervous system disorders	Dizziness, *n* (%)	1 (3.2)	1 (3.2)	0	2 (6.5)
	Dysesthesia, *n* (%)	1 (3.2)	0	0	1 (3.2)
	Headache, *n* (%)	1 (3.2)	0	0	1 (3.2)
	Paresthesia, *n* (%)	2 (6.5)	0	0	2 (6.5)
Vascular disorders	Hypertension, *n* (%)	0	1 (3.2)	0	1 (3.2)
	Hypotension, *n* (%)	2 (6.5)	0	0	2 (6.5)

Adverse events (AEs) and serious AEs are classified according to CTCAE V4.03. Severity and relationship were judged by the investigator. AEs are reported until the primary safety endpoint, i.e., until Visit 7 or Visit 9a for cohorts 1–5 or cohort 6, respectively. For each patient, AEs occurring at least once were counted with the highest CTCAE grading

*CTCAE* common terminology criteria for adverse events, *n* number, *SOC* system organ class, *WBC* white blood cell

Overall, no grade 4 toxicity or serious AEs were observed (Table 2 and Additional file 1: Table S1). There was no dose interruption due to treatment-related AEs; one patient in cohort 6 showed disease progression during study treatment and was discontinued after second dosing.

During long-term safety follow-up until visit 11, no additional safety issues were noticed (Additional file 1: Table S6).

Of the total of 31 patients, six patients (19.3%) had no disease progression at the end of study visit (day 545), whereas twenty-five patients (80.7%) discontinued study follow-up prior to the end of study visit (Additional file 1: Table S7). Median time to follow-up discontinuation for these 25 patients was 86 days (range 22–409) after study drug administration. The reasons for study follow-up discontinuation were evidence of disease progression (MRD progression or hematological relapse, *n* = 17), proceeding to allo-HCT (*n* = 6), lost to follow-up (*n* = 1) and proceeding to alternative treatment (*n* = 1; Additional file 1: Table S7). The six patients proceeding to allo-HCT had a median time on study follow-up of 79 days (range 56–246 days). No patient discontinued study treatment because of AEs related to study drug or death (Additional file 1: Table S7).

### Pharmacokinetics

Pharmacokinetics were assessed in each dose cohort. Peak FLYSYN concentrations were documented 6 h (median) post dose for dose levels ≤ 15 mg/m^2^ (Additional file 1: Table S8). Maximum FLYSYN levels (*C*_max_) were dose proportional and reached 23.1 µg/ml upon single dosing of 45 mg/m^2^. Half-life of FLYSYN was about 7.2 days. Average FLYSYN exposures [area under the curve (AUC)] was dose proportional and similar between dose cohorts 5 and 6 (Additional file 1: Table S8).

### Efficacy

**Fig. 2:**
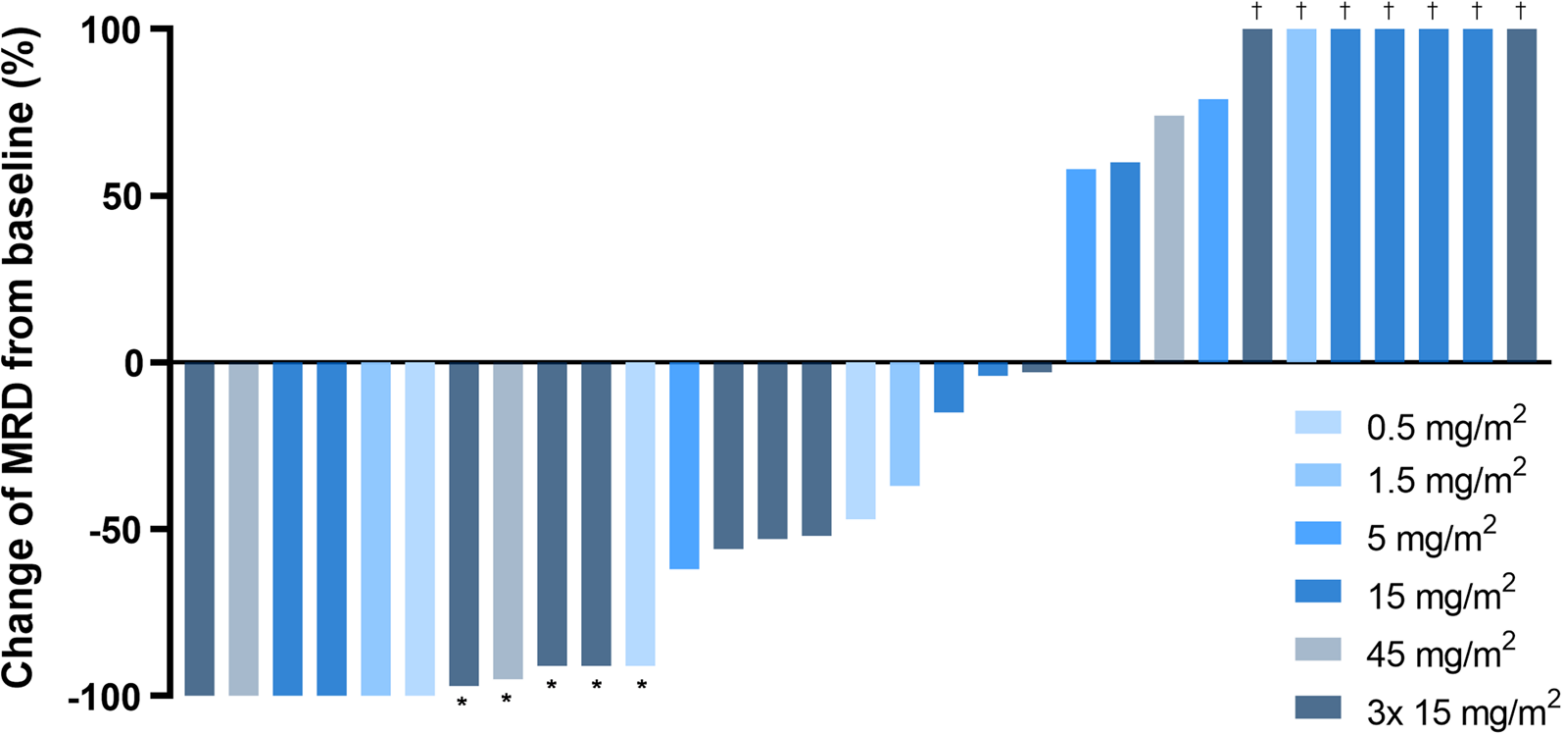
Waterfall plot showing MRD change after FLYSYN treatment. Waterfall plot of the best response after baseline (maximum change of MRD level from baseline) until visit 10 (up to 90 days after first FYSYN application). All dose cohorts are displayed. Six patients were MRD negative, and five patients (asterisk) had a MRD reduction of ≥ 1 log compared to MRD levels prior to FLYSYN treatment. MRD was measured with qPCR or NGS, depending on MRD marker. Seven patients (dagger) did not respond to FLYSYN and had MRD increase > 100%. MRD, minimal residual disease

Efficacy was determined as best response until visit 10 (day 90). In total, 20 (65%) of 31 patients experienced reduction in MRD in BM as defined as any reduction compared to baseline (Fig. 2 and Additional file 1: Table S9). MRD reduction differed for each cohort and was 100%, 67%, 33%, 44%, 67% and 80% for cohorts 1, 2, 3, 4, 5 and 6, respectively. In cohorts 1–4, MRD was reduced in 56% of patients compared to 77% in cohorts 5 and 6. 11/31 (35%) patients achieved an overall molecular response (defined as any > 1 log MRD reduction or negativity in BM) to treatment, with 67%, 33%, 0%, 22%, 67% and 40% in cohorts 1, 2, 3, 4, 5 and 6, respectively. Occurrence of response appeared to be dose-dependent: 28% responses were seen with lower doses compared to 46% in patients receiving 45 mg/m^2^ FLYSYN (single or repetitive dosing). MRD negativity was observed in 19% of all patients until visit 10, with slightly higher numbers in cohorts 1–4 compared to cohorts 5 and 6 (22% vs. 15%), and two patients maintaining documented MRD negativity at end of study (Additional file 1: Table S9). As our trial was designed prior to publication of the current ELN guidelines regarding MRD detection, we separated our patients into MRD_low_ and MRD_high_ groups using the cut-off of 200 NPM1 copies/10,000 ABL copies (2%) according to ELN guidelines [2, 34]. A total of 32% (*n* = 10) of our patients had a NPM1 MRD level below 2%, the other 68% displayed MRD levels above the ELN threshold. Response to treatment was observed more frequently in patients with MRD levels below the cut-off (60% vs. 22%). Of note, the difference in response at least partly leveled out when patients received higher FLYSYN doses, with 60% versus 43% responders upon receiving a total dose of 45 mg/m^2^ compared to 60% vs 9% responders when receiving ≤ 15 mg/m^2^ FLYSYN. Of note, the patients that achieved MRD negativity had presented with median baseline values of 11 NPM1 copies/10,000 ABL copies (Additional file 1: Table S9).

Across the study, median time to MRD response was 29 days (range 15–92). For cohorts 1–4, median time to MRD response was 29 days (range 15–43), whereas in cohorts 5 and 6, MRD response occurred 50.5 days (range 15–92) after FLYSYN treatment. Median time to progression for all treated patients was 6.9 months (95%, CI 3 months–not reached; 0 deaths) (Additional file 1: Fig. S2 and Table S10), with no significant difference observed between cohorts 1–4 and cohorts 5–6. Details are provided in Additional file 1: Appendix.

### Pharmacodynamics

None of the patients developed ADAs (Additional file 1: Table S11). Analysis of potential effects of FLYSYN treatment on stem cell reserve revealed no profound decrease in colony-forming units (Additional file 1: Fig. S3). There was no substantial difference in baseline CD19^+^, CD3^+^, CD3^+^/CD4^+^, CD3^+^/CD8^+^, CD56^+^ and CD14^+^ cell counts with regard to response (Additional file 1: Fig. S4a–f), and neither was there a relevant difference between responders and non-responders regarding percentage and absolute number of NK cells in PB and BM (Additional file 1: Fig. S4g–h). Examination of baseline surface FLT3 expression revealed expression on AML cells in all patients, with no substantial difference according to response (Additional file 1: Fig. S5a–b). Patients not responding to treatment had substantially higher MRD levels at baseline (Additional file 1: Fig. S5c).

## Discussion

This dose-escalation study demonstrates that the Fc-optimized FLT3 antibody FLYSYN applied as monotherapy is very well-tolerated and shows promising clinical activity in terms of achieving MRD reduction or even MRD negativity in AML patients.

Tolerability and safety of FLYSYN was equally distributed over the various dose levels, with a tendency to more AEs at doses > 15 mg/m^2^. As expected, hematologic events constituted the most frequent treatment-related AEs (22·6%), in line with the reportedly low expression of FLT3 in healthy cells of the BM [21]. The mild and transient effect on hematopoiesis, especially neutropenia, was mirrored by the results of CFU assays conducted to monitor for potential BM toxicity showing only mild and transient reduction in colony-forming units. Likewise, most other reported AEs were mild to moderate and mainly comprised flu-like symptoms and fatigue, which may reflect the induced immune activation. No DLTs were recorded.

A major complication of mAb treatment is the development of ADAs, which may neutralize the therapeutic mAb upon repetitive dosing and thus limit efficacy [35]. In none of the patients in our study, development of ADAs against FLYSYN was detected, despite the fact that FLYSYN is a chimerized and not a fully human mAb [22]. Half-life was 7.2 days, which based on preclinical data is sufficient to maintain effective drug levels upon bi-weekly dosing [22, 36].

Molecular response to treatment, defined as ≥ 1 log MRD reduction or negativity in BM, was achieved in 35% of patients, of which two maintained documented MRD negativity at last study visit. Even if efficacy was not the primary endpoint and these findings require further evaluation in consecutive trials, our results are promising, as MRD is associated with disease outcome in AML and also other hematologic malignancies [6, 30, 37–39]. This holds true despite potential technical issues with MRD determination [38, 39] and the fact that in AML, MRD negativity may also occur during the natural course of disease, especially at low MRD levels [2, 32, 34]. In our study, response to treatment was overall associated with lower MRD levels at baseline, and this was likewise observed when patients were grouped in MRD_low_ and MRD_high_ patients according to ELN guidelines. Notably, this effect was less pronounced at higher FLYSYN doses, and overall, a clear trend to higher response rates (46% vs. 28%) was observed for patients treated with higher doses of FLYSYN (cumulative dose > 15 mg/m^2^), which provides important information for further clinical development. Over all applied FLYSYN doses, *c*_max_ serum concentrations above that required for in vitro efficacy were observed [22]. The applied maximum dose of FLYSYN is by far lower than that of rituximab or obinutuzumab applied for B cell lymphoma especially in the maintenance therapy, which mirrors the by far higher expression of CD20 in lymphoma compared to FLT3 in AML in CR with MRD and the accordingly lower required drug level [11, 12, 40].

Together, the favorable safety and preliminary efficacy data confirm the suitability of FLT3 as target antigen for immunotherapy in AML. Notably, activating mutations occurring in the *FLT3* gene, which impact prognosis upon conventional systemic treatment [20], do not affect FLYSYN binding, allowing for application irrespective of *FLT3* mutational status. Overall, there is an increasing interest in targeting FLT3 for immunotherapy not only for mAbs, but also for strategies to induce T cell immunity, like CART cells and bispecific antibodies. Directed to B cell antigens, various CART products and bispecific antibodies are meanwhile approved for lymphoma treatment, with Blinatumomab being first to become available in 2014 [41–46]. In this regard, our data are of interest as they further validate FLT3 as target antigen for myeloid malignancies [47]. Besides immunotherapeutic approaches, hypomethylating agents with or without small molecule drugs are emerging as strategy for the elimination of MRD and are currently under investigation with promising results [48–50]. Limitations of our study include the small number of patients treated across the various FLYSYN dose-level cohorts, the broad range of MRD level at baseline and the fact that the trial was designed before the current ELN guidelines regarding MRD detection were published. In addition, the number of patients who dropped out of the study was higher than anticipated, mainly caused by a stem cell donor becoming available during the study period. To obtain reliable data on efficacy and to identify patients that particularly benefit from FLYSYN treatment, a larger and randomized trial is presently in preparation, which will among others evaluate longer drug exposure/cumulative higher dosing. Nevertheless, our phase I trial documents promising anti-leukemic activity accompanied by a very favorable toxicity profile of our Fc-optimized FLT3 antibody. Considering the beneficial effects reported for demethylating agents after achieving CR and available data that such drugs may boost ADCC induced by mAbs [10, 51, 52], combinatorial application in an upcoming trial may reveal potential synergistic effects and serve to establish FLYSYN as novel immunotherapeutic strategy in AML.

### Supplementary Information

**Additional file 1.** The additional file includes supplementary trial methods, supplementary results (safety laboratory assessment, duration of MRD response), supplementary tables and supplementary figures.

## Data Availability

Data supporting the findings of this study including de-identified patient data are available after final completion of the trial report and are shared according to data sharing guidelines upon reasonable request to the corresponding author HRS.
