# Supplementary material for: Phase I study evaluating the Fc-optimized FLT3 antibody FLYSYN in AML patients with measurable residual disease
Source: J Hematol Oncol. 2023 Aug 17;16:96. doi: 10.1186/s13045-023-01490-w (PMC10433561; doi:10.1186/s13045-023-01490-w)
Supplement: Supplementary file 1 — Additional file 1. The additional file includes supplementary trial methods, supplementary results (safety laboratory assessment, duration of MRD response), supplementary tables and supplementary figures. [file 13045_2023_1490_MOESM1_ESM.docx]

Supplementary Data to the Manuscript Entitled

**Phase I study evaluating the Fc-optimized FLT3 antibody FLYSYN in AML patients with measurable residual disease**

**Table of Contents**

**Supplementary Methods** 2

Detailed inclusion and exclusion criteria for trial participants 2

Additional information on trial methods 2

Trial oversight 2

Statistical considerations including samples size and limiting toxicities 3

Immunophenotyping of peripheral blood cells 3

Colony-forming-unit (CFU) assay 3

Software and statistical analysis 4

**Supplementary Study Results** 5

Safety laboratory assessment 5

Duration of MRD response 5

**Supplementary Tables** 6

Supplementary Table S1: Treatment-emergent adverse events all cohorts 6

Supplementary Table S2: Treatment-emergent adverse events by cohort 8

Supplementary Table S3: Treatment-related adverse events by cohort 11

Supplementary Table S4: Treatment-emergent adverse events by dose of FLYSYN 12

Supplementary Table S5: Treatment-related adverse events by dose of FLYSYN 14

Supplementary Table S6: Treatment-emergent adverse events until visit 11 15

Supplementary Table S7: Reasons for study follow-up discontinuation prior to EOS visit 17

Supplementary Table S8: Pharmacokinetics of FLYSYN by cohort 18

Supplementary Table S9: Response to FLYSYN treatment 19

Supplementary Table S10: Evidence of progressive disease (EPD) free survival after FLYSYN treatment 20

Supplementary Table S11: Development of anti-drug antibodies (ADAs) by cohort 21

Supplementary Table S12: List of prohibited medications within 14 days prior to the first dose of study drug 22

Supplementary Table S13: Detailed information on patients’ prior therapies and remission status 23

**Supplementary Figures** 24

Supplementary Figure S1: Overview of trial design and dosing 24

Supplementary Figure S2: Evidence of progressive disease (EPD) free survival 25

Supplementary Figure S3: Assessment of colony forming units during FLYSYN treatment 26

Supplementary Figure S4: Lymphocyte subsets according to response to FLYSYN treatment. 27

Supplementary Figure S5: FLT3 expression and MRD level according to response to FLYSYN treatment. 28

Supplementary Figure S6: Gating strategy for flow cytometry-based evaluation of immune cells and activation in whole blood samples 29

Supplementary Figure S7: Gating strategy for flow cytometry-based analysis of FLT3 expression on AML cells in the bone marrow 30

**References**  31

# Supplementary Methods

## **Detailed inclusion and exclusion criteria for trial participants**

Eligible participants were men or women aged 18 years or above, who signed the informed consent form. In addition, patients had histologically confirmed acute myeloid leukaemia by WHO criteria^1^, life expectancy of > 3 months, known mutational status for FLT3, willing to receive transfusion of blood products, had an Eastern Cooperative Oncology Group (ECOG) performance status of 0–2. Morphological CR according to European Leukemia Network (ELN) definition^2^ after any therapy except for HSCT with stable or increasing MRD in two sequential measurements using central RT-qPCR and/or NGS constituted the main inclusion criterion. Furthermore, FLT3 expression had to be present on leukemic blasts. All patients had to adhere to adequate contraception methods until six months after study medication, females of child bearing potential had agree to repetitive pregnancy testing.

Exclusion criteria comprised: Pregnant or lactating females; patients proceeding to hematopoietic stem cell transplantation; >5% blasts in bone marrow or extramedullary disease, treatment with a monoclonal antibody within 3 months before study treatment or immunoglobuline intolerance; positivity for human immunodeficieny virus (HIV) or active viral (HAV, HBV or HCV) hepatitis; no consent for biobanking or processing of individual patient related data; presence of any medical/psychiatric condition or laboratory abnormalities which may limit full compliance to the study; Prior history of malignancies, other than AML/myelodysplastic syndrome (MDS), unless the subject has been free of the disease for ≥ 2 years (exceptions include the following: Basal cell carcinoma of the skin, carcinoma in situ of the cervix, carcinoma in situ of the breast, histological finding of prostate cancer of TNM stage T1); patients receiving any medication listed in Supplementary Table S12; uncontrolled infection; current treatment with immunosuppressive agents; patients under ongoing treatment with another investigational medication or having been treated with an investigational medication within 14 days of screening; or systemic diseases (cardiovascular, renal, hepatic, etc.) that would prevent study treatment (e.g., creatinine >1.5x upper normal serum level; bilirubin, AST or AP >2.5x upper normal serum level; heart failure NYHA III/IV; severe obstructive or restrictive ventilation disorder).

In case of violation of in- or exclusion criteria, e.g. MRD negativity at screening, re-screening was allowed.

## **Additional information on trial methods**

Full blood counts and chemistry were analyzed on days 1–3, 8, 15, 22, 29, 36, 43, 90 and thereafter every 30 days after administration of the first scheduled FLYSYN dose. For all cohorts, blood samples for pharmacokinetic and cytokine assessments were collected at hours 0, 1.5, 3, 6, and 18 post-dose on day 1 and 2. For cohort 6, blood samples for pharmacokinetic and cytokine assessments were additionally collected at hours 0, 1.5, 3, and 6 post-dose on day 15 and 29. Non-compartmental methods were used to determine FLYSYN pharmacokinetic parameters including maximum observed plasma concentration, time to reach this concentration, and area under the plasma concentration-time curve (AUC) from 0 h to 24 h dose interval. The maximum tested dose was selected taking into consideration ex vivo data obtained from AML patients which had been treated with FLYSYN on “compassionate need basis” during an “individualized experimental therapy regime” (pursuant to section 13 of the German Medicines Act (AMG) and in accordance with the principles of the WMA Declaration of Helsinki (section 37) and the department of health and human services Belmont report) prior to initiation of this trial. Saturated binding to FLT3 on leukemic blasts was observed after application of 10mg FLYSYN (data not shown). Bone marrow (BM) aspirate and biopsies were taken at screening, at days 1, 15, 22 (except for cohort 6: day 29), 43, 90, 180 and 365 for anti-leukemic activity assessments, which were done at the investigator sites as per the International Working Group criteria for acute myeloid leukemia.^3^ Per investigator discretion, additional BM samples were collected if there was peripheral blood count recovery suggestive of an improved response or in patients at risk of relapsed or resistant disease. Peripheral blood (PB) or BM specimens, or both, were collected from patients at baseline and consecutive visits for biomarker analysis, which was an exploratory endpoint. MRD assessment was conducted by central laboratory according to standard procedure (University Hospital Ulm and Medizinische Hochschule Hannover for qPCR and NGS, respectively).^4,5^ For qPCR, copy of mutated NPM1/10,000 ABL1 copies were provided. Absolute MRD threshold used in the study to define MRD positivity: NPM1/ABLx10^4^ > 0 = positive. Sensitivity depends on ABL values ranging between 10^-5^ and 10^-6^. For NGS, MRD positivity was defined as any MRD above a threshold of 0.01%, which had been validated in a prior study,^4^ and sensitivity of NGS was 10^-4^. MRD markers detectable by NGS were monitored by this method during conduct of the trial based on the possibility to detect and monitor different aberrations in parallel, despite the fact that as of now, qPCR constitutes the standard for monitoring of RUNX1-RUNX1T1. If patients had more than one MRD marker, the MRD marker assessable by qPCR was used for efficacy analysis. FLT3 expression on leukemic blasts as inclusion criterion was confirmed by flow cytometry.^6^ Status of FLT3 mutation was assessed at diagnosis per institutional guidelines and provided.

## **Trial oversight**

Synimmune GmbH, the sponsor, provided financial support for the trial and participated in the design, trial conduct, analysis, and interpretation of the data. All the authors had full access to the data, signed confidentiality agreements with the sponsors regarding the data, and vouch for the completeness and accuracy of the data and analyses and for the fidelity of the trial to the protocol. The first draft of the manuscript was written by the first and corresponding author, with input from all the authors. All the authors critically reviewed and provided feedback on all subsequent versions of the manuscript. The trial was conducted in accordance with the Declaration of Helsinki and the Good Clinical Practice guidelines of the International Council for Harmonisation. The protocol and related documents were approved by the responsible federal agency and applicable ethics committees, and all the patients provided written informed consent. An independent data and safety monitoring committee reviewed safety data after each cohort and provided recommendations for continuation or termination of the trial.

## **Statistical considerations including sample size and limiting toxicities**

Dose escalation follows a standard 3+3 design with a minimum of three evaluable patients enrolled per cohort. The total number of patients depends on the numbers of tested dose levels (DL`s) and patients per DL. Assuming that no dose limiting toxicity occurs, the estimated maximum number of patients in the study is 31. To assess preliminary efficacy of FLYSYN at least 19 patients will be treated in at least one cohort (either Cohort 1 to 4) or combined in Cohorts 4 to 6 to assess efficacy. For evaluating efficacy, molecular response is used, which is defined as MRD negativity (i.e. no detectable AML-MRD marker gene) or one log reduction of at least one AML-MRD marker gene at visit 9 (and a sensitivity analysis using best response until V10). The null hypothesis H_0_ is defined as a molecular response rate equal of p_0_=0.2 whereas the (interesting) alternative used for power calculation is a molecular response rate of p_0_=0.5. The sample size is calculated such that the target power of 90 % is reached while controlling the type I error rate of 10%. In particular, for n = 19 patients, the power of the design is 91.6% and the type I error rate is 6.8%.

In this clinical trial the following predefined rules for dose limiting toxicities (DLT) were implemented:

Laboratory and AE toxicities are graded according to NCI CTCAE, version 4.03, with DLT being defined as an AE or abnormal laboratory value assessed as having a suspected or unknown relationship to the study drug (FLYSYN) and meeting one of the following criteria:

Hematologic DLT:

- Grade 4 thrombocytopenia, anemia, neutropenia or respective decrease in any other sub-fraction of white lineage that does not resolve to grade 2 or less within 14 days unless attributable to AML progression.

Non-hematologic DLT:

- All other events ≥ grade 3 (excluding hypersensitivity reactions and fatigue) unless attributable to AML progression.

All other treatment-related grade 4 events:

- In addition, any event will only qualify as DLT if a worsening of the event from baseline of at least 2 CTC grades occurred unless attributable to AML progression.

## **Immunophenotyping of peripheral blood cells**

Peripheral blood (100 µL) was stained with CD8a-BV605 (RPA-T8, 1:100), CD3-APC/Fire (SK7, 1:100), CD56-PECy7 (5.1H11, 1:25), CD14-FITC (M5E2, 1:25), CD4-Pacific Blue (RPA-T4, 1:50), CD19-BV510 (HIB19, 1:100) (all from Biolegend), mIgG1-APC (MOPC-21, 1:100), mIgG1-PE (MOPC-21, 1:10) (all from BD Bioscience) for 15 min at 21°C and subsequently 100 µL of OptiLyse C solution (Beckman Coulter) was added for additional 20 min. Finally, 1 mL of distilled water was added for 15 min followed by centrifugation at 300xg for 5 min. After removing the supernatant, cells were resuspended in 1 mL PBS followed by one additional centrifugation step. Cells were diluted in 100 µL PBS and data acquisition was performed with a BD FACSLyric system (BD Bioscience) and analysis was done with FlowJo_V10 software (BD Bioscience). Exemplary gating strategies are displayed in Supplementary Figures S6 and S7.

## **Colony-forming-unit (CFU) assay**

Mononuclear cells of bone marrow aspirates were isolated with Biocoll Separating Solution (Biochrom) by density gradient centrifugation. Cells were viably stored in freezing media containing RPMI160 (Gibco, Thermofisher) + 20% FCS (Biochrom) + 10% DMSO (Sigma) in liquid nitrogen. For CFU assays cells were thawed in culture media containing IMDM (Gibco, Thermofisher) + 10% FCS. After counting 50,000 cells in 50 µl culture media per sample (3x replicates) were resuspended in 2 mL MethoCult media (StemCell Technologies) and plated in 35 mm perti dishes. Plates were incubated at 37°C and 5% CO_2_ for 14 days followed by counting of BFU-E, CFU-GM and CFU-GEMM colonies. Combined score of all colonies was used for analysis.

## **Software and statistical analysis**

Flow cytometric data was analyzed using FlowJo 10.7.1 (BD). Graphs were plotted using GraphPad Prism 9.2.0. Statistical analyses were conducted using SAS Version 9.4.

# Supplementary Study Results

## **Safety laboratory assessment**

Safety laboratory AEs were graded according to a CTCAE V4.03 grading scale. Abnormal laboratory parameters were assessed by the investigators for clinical significance and reported. If normal at baseline, deviations from normal range were reported for 26 parameters (natrium, potassium, phosphate, creatinine, urea, uric acid, aspartate transaminase, C-reactive protein, bilirubin, serum protein, creatine kinase, alanine transaminase, alkaline phosphatase, lactate dehydrogenase, amylase, lipase, blood glucosis, prothrombin time, hemoglobulin, thrombocytes, leukocytes, neutrophile count, eosinophile count, basophilie count, lymphocyte count and monocyte count) until V7 or V9a for cohorts 1-5 or cohort 6, respectively. Among the most frequent deviations, C-reactive protein elevation was reported in 14 patients (45%).

## **Duration of MRD response**

In patients responding to FLYSYN treatment, duration of response differed largely with a median of 70.5 days (95% CI: 8-157 days). Of note, two patients, converting to MRD negativity before visit 10, remained MRD negative at 545 days (last study assessment).

**Supplementary Tables**

## **Supplementary Table S1:** Treatment-emergent adverse events all cohorts

|  | | **All subjects (n=31)** | | | |
| --- | --- | --- | --- | --- | --- |
| **System organ class** | **CTCAE term** | **Grade 1** | **Grade 2** | **Grade 3** | **Any grade** |
| Patients with events | All terms | 15 (48.4) | 7 (22.6) | 3 (9.7) | 25 (81) |
| Blood and lymphatic system disorders | Anemia, n (%) | 5 (16.1) | 1 (3.2) | 0 | 6 (19.4) |
| Cardiac disorders | Left ventricular systolic dysfunction, n (%) | 0 | 1 (3.2) | 0 | 1 (3.2) |
| Gastrointestinal disorders | Constipation, n (%) | 2 (6.5) | 0 | 0 | 2 (6.5) |
|  | Diarrhea, n (%) | 1 (3.2) | 0 | 0 | 1 (3.2) |
|  | Dry mouth, n (%) | 1 (3.2) | 0 | 0 | 1 (3.2) |
|  | Gastrointestinal pain, n (%) | 1 (3.2) | 0 | 0 | 1 (3.2) |
|  | Nausea, n (%) | 1 (3.2) | 0 | 0 | 1 (3.2) |
|  | Oral dysesthesia, n (%) | 1 (3.2) | 0 | 0 | 1 (3.2) |
| General disorders and administration site conditions | Chills, n (%) | 3 (9.7) | 0 | 0 | 3 (9.7) |
|  | Edema limbs, n (%) | 1 (3.2) | 0 | 0 | 1 (3.2) |
|  | Fatigue, n (%) | 4 (12.9) | 0 | 0 | 4 (12.9) |
|  | Fever, n (%) | 3 (9.7) | 0 | 0 | 3 (9.7) |
|  | Flu like symptoms, n (%) | 2 (6.5) | 0 | 0 | 2 (6.5) |
|  | General disorders and administration site conditions - Other, night sweats, n (%) | 1 (3.2) | 0 | 0 | 1 (3.2) |
|  | Non-cardiac chest pain, n (%) | 1 (3.2) | 0 | 0 | 1 (3.2) |
| Infections and infestations | Catheter related infection, n (%) | 1 (3.2) | 0 | 0 | 1 (3.2) |
| Investigations | Alanine aminotransferase increased, n (%) | 1 (3.2) | 0 | 0 | 1 (3.2) |
|  | Blood bilirubin increased, n (%) | 2 (6.5) | 0 | 0 | 2 (6.5) |
|  | CPK increased, n (%) | 1 (3.2) | 0 | 0 | 1 (3.2) |
|  | Creatinine increased, n (%) | 1 (3.2) | 0 | 0 | 1 (3.2) |
|  | Electrocardiogram QT corrected interval prolonged, n (%) | 1 (3.2) | 0 | 0 | 1 (3.2) |
|  | INR increased, n (%) | 1 (3.2) | 0 | 0 | 1 (3.2) |
|  | Investigations - Other, n (%) | 3 (9.7) | 0 | 0 | 3 (9.7) |
|  | Lipase increased, n (%) | 0 | 1 (3.2) | 0 | 1 (3.2) |
|  | Lymphocyte count decreased, n (%) | 3 (9.7) | 1 (3.2) | 0 | 4 (12.9) |
|  | Neutrophil count decreased, n (%) | 3 (9.7) | 3 (9.7) | 2 (6.5) | 8 (25.8) |
|  | Platelet count decreased, n (%) | 2 (6.5) | 0 | 0 | 2 (6.5) |
|  | Serum amylase increased, n (%) | 0 | 2 (6.5) | 0 | 2 (6.5) |
|  | White blood cell decreased, n (%) | 3 (9.7) | 3 (9.7) | 0 | 6 (19.4) |
| Metabolism and nutrition disorders | Hyperkalemia, n (%) | 1 (3.2) | 0 | 0 | 1 (3.2) |
|  | Hyperuricemia, n (%) | 2 (6.5) | 0 | 0 | 2 (6.5) |
|  | Hypokalemia, n (%) | 1 (3.2) | 1 (3.2) | 0 | 2 (6.5) |
|  | Hyponatremia, n (%) | 1 (3.2) | 0 | 0 | 1 (3.2) |
| Musculoskeletal and connective tissue disorders | Back pain, n (%) | 1 (3.2) | 0 | 1 (3.2) | 2 (6.5) |
|  | Musculoskeletal and connective tissue disorder - Other, n (%) | 1 (3.2) | 0 | 0 | 1 (3.2) |
|  | Pain in extremity, n (%) | 3 (9.7) | 1 (3.2) | 0 | 4 (12.9) |
| Nervous system disorders | Dizziness, n (%) | 1 (3.2) | 1 (3.2) | 0 | 2 (6.5) |
|  | Dysesthesia, n (%) | 1 (3.2) | 0 | 0 | 1 (3.2) |
|  | Headache, n (%) | 1 (3.2) | 0 | 0 | 1 (3.2) |
|  | Paresthesia, n (%) | 2 (6.5) | 0 | 0 | 2 (6.5) |
|  | Peripheral sensory neuropathy, n (%) | 1 (3.2) | 0 | 0 | 1 (3.2) |
| Psychiatric disorders | Anxiety, n (%) | 1 (3.2) | 0 | 0 | 1 (3.2) |
| Respiratory, thoracic and mediastinal disorders | Cough, n (%) | 1 (3.2) | 0 | 0 | 1 (3.2) |
|  | Sore throat, n (%) | 1 (3.2) | 0 | 0 | 1 (3.2) |
| Skin and subcutaneous tissue disorders | Dry skin, n (%) | 1 (3.2) | 0 | 0 | 1 (3.2) |
| Vascular disorders | Hypertension, n (%) | 0 | 2 (6.5) | 0 | 2 (6.5) |
|  | Hypotension, n (%) | 2 (6.5) | 0 | 0 | 2 (6.5) |

Adverse events (AEs) and serious AEs are classified according to CTCAE V4.03. Severity and relationship were judged by the investigator. AEs are reported until the primary safety endpoint, i.e. until V7 or V9a for cohorts 1-5 or cohort 6, respectively. For each patient the adverse event occurring at least once was counted with the highest CTCAE grading. For “Investigations – Other”, interleukin 2 receptor increased, ferritin increased and C-reactive protein increased were reported. For “Musculoskeletal and connective tissue disorder – Other” whole body pain and snapping finder (left thumb) were reported. CTCAE, Common Terminology Criteria for Adverse Events; n, number; SOC, system organ class; WBC, white blood cell

## **Supplementary Table S2:** Treatment-emergent adverse events by cohort

|  | | **Cohort 1 (n=3)** | | | **Cohort 2 (n=3)** | | | **Cohort 3 (n=3)** | | | **Cohort 4 (n=9)** | | | **Cohort 5 (n=3)** | | | **Cohort 6 (n=10)** | | |
| --- | --- | --- | --- | --- | --- | --- | --- | --- | --- | --- | --- | --- | --- | --- | --- | --- | --- | --- | --- |
| **System organ class** | **CTCAE term** | **Grade 1** | **Grade 2** | **Grade 3** | **Grade 1** | **Grade 2** | **Grade 3** | **Grade 1** | **Grade 2** | **Grade 3** | **Grade 1** | **Grade 2** | **Grade 3** | **Grade 1** | **Grade 2** | **Grade 3** | **Grade 1** | **Grade 2** | **Grade 3** |
| Blood and lymphatic system disorders | Anemia, n (%) | 1 (33.3) | 0 | 0 | 0 | 0 | 0 | 0 | 0 | 0 | 1 (11.1) | 0 | 0 | 0 | 0 | 0 | 3 (30.0) | 1 (10.0) | 0 |
| Cardiac disorders | Left ventricular systolic dysfunction, n (%) | 0 | 0 | 0 | 0 | 0 | 0 | 0 | 1 (33.3) | 0 | 0 | 0 | 0 | 0 | 0 | 0 | 0 | 0 | 0 |
| Gastrointestinal disorders | Constipation, n (%) | 0 | 0 | 0 | 0 | 0 | 0 | 1 (33.3) | 0 | 0 | 1 (11.1) | 0 | 0 | 0 | 0 | 0 | 0 | 0 | 0 |
|  | Diarrhea, n (%) | 0 | 0 | 0 | 0 | 0 | 0 | 0 | 0 | 0 | 0 | 0 | 0 | 0 | 0 | 0 | 1 (10.0) | 0 | 0 |
|  | Dry mouth, n (%) | 0 | 0 | 0 | 0 | 0 | 0 | 1 (33.3) | 0 | 0 | 0 | 0 | 0 | 0 | 0 | 0 | 0 | 0 | 0 |
|  | Gastrointestinal pain, n (%) | 0 | 0 | 0 | 0 | 0 | 0 | 0 | 0 | 0 | 0 | 0 | 0 | 0 | 0 | 0 | 1 (10.0) | 0 | 0 |
|  | Nausea, n (%) | 0 | 0 | 0 | 0 | 0 | 0 | 0 | 0 | 0 | 0 | 0 | 0 | 0 | 0 | 0 | 1 (10.0) | 0 | 0 |
|  | Oral dysesthesia, n (%) | 0 | 0 | 0 | 0 | 0 | 0 | 0 | 0 | 0 | 0 | 0 | 0 | 0 | 0 | 0 | 1 (10.0) | 0 | 0 |
| General disorders and administration site conditions | Chills, n (%) | 0 | 0 | 0 | 0 | 0 | 0 | 0 | 0 | 0 | 1 (11.1) | 0 | 0 | 0 | 0 | 0 | 2 (20.0) | 0 | 0 |
|  | Edema limbs, n (%) | 0 | 0 | 0 | 0 | 0 | 0 | 0 | 0 | 0 | 0 | 0 | 0 | 0 | 0 | 0 | 1 (10.0) | 0 | 0 |
|  | Fatigue, n (%) | 0 | 0 | 0 | 0 | 0 | 0 | 0 | 0 | 0 | 0 | 0 | 0 | 1 (33.3) | 0 | 0 | 3 (30.0) | 0 | 0 |
|  | Fever, n (%) | 0 | 0 | 0 | 0 | 0 | 0 | 0 | 0 | 0 | 1 (11.1) | 0 | 0 | 1 (33.3) | 0 | 0 | 1 (10.0) | 0 | 0 |
|  | Flu like symptoms, n (%) | 0 | 0 | 0 | 0 | 0 | 0 | 0 | 0 | 0 | 0 | 0 | 0 | 0 | 0 | 0 | 2 (20.0) | 0 | 0 |
|  | General disorders and administration site conditions - Other, night sweats, n (%) | 0 | 0 | 0 | 0 | 0 | 0 | 0 | 0 | 0 | 0 | 0 | 0 | 1 (33.3) | 0 | 0 | 0 | 0 | 0 |
|  | Non-cardiac chest pain, n (%) | 0 | 0 | 0 | 0 | 0 | 0 | 0 | 0 | 0 | 0 | 0 | 0 | 0 | 0 | 0 | 1 (10.0) | 0 | 0 |
| Infections and infestations | Catheter related infection, n (%) | 0 | 0 | 0 | 0 | 0 | 0 | 1 (33.3) | 0 | 0 | 0 | 0 | 0 | 0 | 0 | 0 | 0 | 0 | 0 |
| Investigations | Alanine aminotransferase increased, n (%) | 0 | 0 | 0 | 0 | 0 | 0 | 0 | 0 | 0 | 0 | 0 | 0 | 0 | 0 | 0 | 1 (10.0) | 0 | 0 |
|  | Blood bilirubin increased, n (%) | 0 | 0 | 0 | 0 | 0 | 0 | 0 | 0 | 0 | 0 | 0 | 0 | 1 (33.3) | 0 | 0 | 1 (10.0) | 0 | 0 |
|  | CPK increased, n (%) | 0 | 0 | 0 | 0 | 0 | 0 | 0 | 0 | 0 | 0 | 0 | 0 | 0 | 0 | 0 | 1 (10.0) | 0 | 0 |
|  | Creatinine increased, n (%) | 1 (33.3) | 0 | 0 | 0 | 0 | 0 | 0 | 0 | 0 | 0 | 0 | 0 | 0 | 0 | 0 | 0 | 0 | 0 |
|  | Electrocardiogram QT corrected interval prolonged, n (%) | 1 (33.3) | 0 | 0 | 0 | 0 | 0 | 0 | 0 | 0 | 0 | 0 | 0 | 0 | 0 | 0 | 0 | 0 | 0 |
|  | INR increased, n (%) | 0 | 0 | 0 | 0 | 0 | 0 | 0 | 0 | 0 | 0 | 0 | 0 | 0 | 0 | 0 | 1 (10.0) | 0 | 0 |
|  | Investigations - Other, n (%) | 0 | 0 | 0 | 0 | 0 | 0 | 0 | 0 | 0 | 1 (11.1) | 0 | 0 | 0 | 0 | 0 | 2 (20.0) | 0 | 0 |
|  | Lipase increased, n (%) | 0 | 1 (33.3) | 0 | 0 | 0 | 0 | 0 | 0 | 0 | 0 | 0 | 0 | 0 | 0 | 0 | 0 | 0 | 0 |
|  | Lymphocyte count decreased, n (%) | 0 | 0 | 0 | 0 | 0 | 0 | 0 | 0 | 0 | 0 | 0 | 0 | 0 | 1 (33.3) | 0 | 3 (30.0) | 0 | 0 |
|  | Neutrophil count decreased, n (%) | 0 | 0 | 0 | 0 | 0 | 0 | 0 | 0 | 0 | 1 (11.1) | 0 | 1 (11.1) | 0 | 1 (33.3) | 0 | 2 (20.0) | 2 (20.0) | 1 (10.0) |
|  | Platelet count decreased, n (%) | 0 | 0 | 0 | 0 | 0 | 0 | 0 | 0 | 0 | 2 (22.2) | 0 | 0 | 0 | 0 | 0 | 0 | 0 | 0 |
|  | Serum amylase increased, n (%) | 0 | 1 (33.3) | 0 | 0 | 0 | 0 | 0 | 0 | 0 | 0 | 0 | 0 | 0 | 0 | 0 | 0 | 1 (10.0) | 0 |
|  | White blood cell decreased, n (%) | 0 | 0 | 0 | 0 | 0 | 0 | 0 | 0 | 0 | 0 | 0 | 0 | 0 | 1 (33.3) | 0 | 3 (30.0) | 2 (20.0) | 0 |
| Metabolism and nutrition disorders | Hyperkalemia, n (%) | 0 | 0 | 0 | 0 | 0 | 0 | 0 | 0 | 0 | 0 | 0 | 0 | 0 | 0 | 0 | 1 (10.0) | 0 | 0 |
|  | Hyperuricemia, n (%) | 0 | 0 | 0 | 1 (33.3) | 0 | 0 | 0 | 0 | 0 | 0 | 0 | 0 | 0 | 0 | 0 | 1 (10.0) | 0 | 0 |
|  | Hypokalemia, n (%) | 0 | 0 | 0 | 0 | 0 | 0 | 0 | 0 | 0 | 1 (11.1) | 1 (11.1) | 0 | 0 | 0 | 0 | 0 | 0 | 0 |
|  | Hyponatremia, n (%) | 0 | 0 | 0 | 0 | 0 | 0 | 0 | 0 | 0 | 0 | 0 | 0 | 0 | 0 | 0 | 1 (10.0) | 0 | 0 |
| Musculoskeletal and connective tissue disorders | Back pain, n (%) | 0 | 0 | 0 | 0 | 0 | 0 | 0 | 0 | 0 | 0 | 0 | 0 | 1 (33.3) | 0 | 0 | 0 | 0 | 1 (10.0) |
|  | Musculoskeletal and connective tissue disorder - Other, n (%) | 0 | 0 | 0 | 0 | 0 | 0 | 1 (33.3) | 0 | 0 | 0 | 0 | 0 | 0 | 0 | 0 | 0 | 0 | 0 |
|  | Pain in extremity, n (%) | 0 | 0 | 0 | 0 | 0 | 0 | 0 | 0 | 0 | 0 | 0 | 0 | 2 (66.7) | 0 | 0 | 1 (10.0) | 1 (10.0) | 0 |
| Nervous system disorders | Dizziness, n (%) | 0 | 0 | 0 | 0 | 0 | 0 | 0 | 0 | 0 | 0 | 0 | 0 | 0 | 0 | 0 | 1 (10.0) | 1 (10.0) | 0 |
|  | Dysesthesia, n (%) | 0 | 0 | 0 | 0 | 0 | 0 | 0 | 0 | 0 | 0 | 0 | 0 | 0 | 0 | 0 | 1 (10.0) | 0 | 0 |
|  | Headache, n (%) | 0 | 0 | 0 | 0 | 0 | 0 | 0 | 0 | 0 | 0 | 0 | 0 | 0 | 0 | 0 | 1 (10.0) | 0 | 0 |
|  | Paresthesia, n (%) | 0 | 0 | 0 | 0 | 0 | 0 | 0 | 0 | 0 | 1 (11.1) | 0 | 0 | 0 | 0 | 0 | 1 (10.0) | 0 | 0 |
|  | Peripheral sensory neuropathy, n (%) | 0 | 0 | 0 | 0 | 0 | 0 | 0 | 0 | 0 | 0 | 0 | 0 | 0 | 0 | 0 | 1 (10.0) | 0 | 0 |
| Psychiatric disorders | Anxiety, n (%) | 0 | 0 | 0 | 0 | 0 | 0 | 0 | 0 | 0 | 1 (11.1) | 0 | 0 | 0 | 0 | 0 | 0 | 0 | 0 |
| Respiratory, thoracic and mediastinal disorders | Cough, n (%) | 0 | 0 | 0 | 0 | 0 | 0 | 0 | 0 | 0 | 1 (11.1) | 0 | 0 | 0 | 0 | 0 | 0 | 0 | 0 |
|  | Sore throat, n (%) | 0 | 0 | 0 | 0 | 0 | 0 | 0 | 0 | 0 | 0 | 0 | 0 | 1 (33.3) | 0 | 0 | 0 | 0 | 0 |
| Skin and subcutaneous tissue disorders | Dry skin, n (%) | 0 | 0 | 0 | 0 | 0 | 0 | 0 | 0 | 0 | 0 | 0 | 0 | 1 (33.3) | 0 | 0 | 0 | 0 | 0 |
| Vascular disorders | Hypertension, n (%) | 0 | 0 | 0 | 0 | 0 | 0 | 0 | 0 | 0 | 0 | 2 (22.2) | 0 | 0 | 0 | 0 | 0 | 0 | 0 |
|  | Hypotension, n (%) | 1 (33.3) | 0 | 0 | 0 | 0 | 0 | 0 | 0 | 0 | 0 | 0 | 0 | 0 | 0 | 0 | 1 (10.0) | 0 | 0 |

Adverse events (AEs) and serious AEs are classified according to CTCAE V4.03. Severity and relationship were judged by the investigator. AEs of each cohort are displayed. Adverse events are reported until the primary safety endpoint, i.e. until V7 or V9a for cohorts 1-5 or cohort 6, respectively. For each patient the adverse event occurring at least once was counted with the highest CTCAE grading. For “Investigations – Other”, interleukin 2 receptor increased, ferritin increased and C-reactive protein increased were reported. For “Musculoskeletal and connective tissue disorder – Other” whole body pain and snapping finder (left thumb) were reported. CTCAE, Common Terminology Criteria for Adverse Events; n, number; SOC, system organ class; WBC, white blood cell

## **Supplementary Table S3: Treatment-related adverse events by cohort**

|  | | **Cohort 1 (n=3)** | | | **Cohort 2 (n=3)** | | | **Cohort 3 (n=3)** | | | **Cohort 4 (n=9)** | | | **Cohort 5 (n=3)** | | | **Cohort 6 (n=10)** | | |
| --- | --- | --- | --- | --- | --- | --- | --- | --- | --- | --- | --- | --- | --- | --- | --- | --- | --- | --- | --- |
| **System organ class** | **CTCAE term** | **Grade 1** | **Grade 2** | **Grade 3** | **Grade 1** | **Grade 2** | **Grade 3** | **Grade 1** | **Grade 2** | **Grade 3** | **Grade 1** | **Grade 2** | **Grade 3** | **Grade 1** | **Grade 2** | **Grade 3** | **Grade 1** | **Grade 2** | **Grade 3** |
| Blood and lymphatic system disorders | Anemia, n (%) | 1 (33.3) | 0 | 0 | 0 | 0 | 0 | 0 | 0 | 0 | 1 (11.1) | 0 | 0 | 0 | 0 | 0 | 3 (30.0) | 1 (10.0) | 0 |
| Gastrointestinal disorders | Nausea, n (%) | 0 | 0 | 0 | 0 | 0 | 0 | 0 | 0 | 0 | 0 | 0 | 0 | 0 | 0 | 0 | 1 (10.0) | 0 | 0 |
| General disorders and administration site conditions | Chills, n (%) | 0 | 0 | 0 | 0 | 0 | 0 | 0 | 0 | 0 | 1 (11.1) | 0 | 0 | 0 | 0 | 0 | 1 (10.0) | 0 | 0 |
|  | Edema limbs, n (%) | 0 | 0 | 0 | 0 | 0 | 0 | 0 | 0 | 0 | 0 | 0 | 0 | 0 | 0 | 0 | 1 (10.0) | 0 | 0 |
|  | Fatigue, n (%) | 0 | 0 | 0 | 0 | 0 | 0 | 0 | 0 | 0 | 0 | 0 | 0 | 0 | 0 | 0 | 3 (30.0) | 0 | 0 |
|  | Fever, n (%) | 0 | 0 | 0 | 0 | 0 | 0 | 0 | 0 | 0 | 1 (11.1) | 0 | 0 | 1 (33.3) | 0 | 0 | 1 (10.0) | 0 | 0 |
|  | Flu like symptoms, n (%) | 0 | 0 | 0 | 0 | 0 | 0 | 0 | 0 | 0 | 0 | 0 | 0 | 0 | 0 | 0 | 2 (20.0) | 0 | 0 |
|  | Non-cardiac chest pain, n (%) | 0 | 0 | 0 | 0 | 0 | 0 | 0 | 0 | 0 | 0 | 0 | 0 | 0 | 0 | 0 | 1 (10.0) | 0 | 0 |
| Investigations | Blood bilirubin increased, n (%) | 0 | 0 | 0 | 0 | 0 | 0 | 0 | 0 | 0 | 0 | 0 | 0 | 1 (33.3) | 0 | 0 | 1 (10.0) | 0 | 0 |
|  | Investigations - Other, interleukine 2 receptor increased, n (%) | 0 | 0 | 0 | 0 | 0 | 0 | 0 | 0 | 0 | 0 | 0 | 0 | 0 | 0 | 0 | 1 (10.0) | 0 | 0 |
|  | Lymphocyte count decreased, n (%) | 0 | 0 | 0 | 0 | 0 | 0 | 0 | 0 | 0 | 0 | 0 | 0 | 0 | 1 (33.3) | 0 | 3 (30.0) | 0 | 0 |
|  | Neutrophil count decreased, n (%) | 0 | 0 | 0 | 0 | 0 | 0 | 0 | 0 | 0 | 0 | 0 | 1 (11.1) | 0 | 1 (33.3) | 0 | 2 (20.0) | 2 (20.0) | 1 (10.0) |
|  | Platelet count decreased, n (%) | 0 | 0 | 0 | 0 | 0 | 0 | 0 | 0 | 0 | 2 (22.2) | 0 | 0 | 0 | 0 | 0 | 0 | 0 | 0 |
|  | WBC decreased, n (%) | 0 | 0 | 0 | 0 | 0 | 0 | 0 | 0 | 0 | 0 | 0 | 0 | 0 | 1 (33.3) | 0 | 3 (30.0) | 2 (20.0) | 0 |
| Musculoskeletal and connective tissue disorders | Back pain, n (%) | 0 | 0 | 0 | 0 | 0 | 0 | 0 | 0 | 0 | 0 | 0 | 0 | 0 | 0 | 0 | 0 | 0 | 1 (10.0) |
|  | Pain in extremity, n (%) | 0 | 0 | 0 | 0 | 0 | 0 | 0 | 0 | 0 | 0 | 0 | 0 | 0 | 0 | 0 | 1 (10.0) | 1 (10.0) | 0 |
| Nervous system disorders | Dizziness, n (%) | 0 | 0 | 0 | 0 | 0 | 0 | 0 | 0 | 0 | 0 | 0 | 0 | 0 | 0 | 0 | 1 (10.0) | 1 (10.0) | 0 |
|  | Dysesthesia, n (%) | 0 | 0 | 0 | 0 | 0 | 0 | 0 | 0 | 0 | 0 | 0 | 0 | 0 | 0 | 0 | 1 (10.0) | 0 | 0 |
|  | Headache, n (%) | 0 | 0 | 0 | 0 | 0 | 0 | 0 | 0 | 0 | 0 | 0 | 0 | 0 | 0 | 0 | 1 (10.0) | 0 | 0 |
|  | Paresthesia, n (%) | 0 | 0 | 0 | 0 | 0 | 0 | 0 | 0 | 0 | 1 (11.1) | 0 | 0 | 0 | 0 | 0 | 1 (10.0) | 0 | 0 |
| Vascular disorders | Hypertension, n (%) | 0 | 0 | 0 | 0 | 0 | 0 | 0 | 0 | 0 | 0 | 1 (11.1) | 0 | 0 | 0 | 0 | 0 | 0 | 0 |
|  | Hypotension, n (%) | 1 (33.3) | 0 | 0 | 0 | 0 | 0 | 0 | 0 | 0 | 0 | 0 | 0 | 0 | 0 | 0 | 1 (10.0) | 0 | 0 |

Adverse events (AEs) and serious AEs are classified according to CTCAE V4.03. Severity and relationship were judged by the investigator. AEs of each cohort are displayed. Adverse events are reported until the primary safety endpoint, i.e. until V7 or V9a for cohorts 1-5 or cohort 6, respectively. For each patient the adverse event occurring at least once was counted with the highest CTCAE grading. CTCAE, Common Terminology Criteria for Adverse Events; n, number; SOC, system organ class; WBC, white blood cell.

## **Supplementary Table S4:** Treatment-emergent adverse events by dose of FLYSYN

|  | | **Cohorts 1-4 (n=18)** | | | | **Cohorts 5-6 (n=13)** | | | |
| --- | --- | --- | --- | --- | --- | --- | --- | --- | --- |
| **System organ class** | **CTCAE term** | **Grade 1** | **Grade 2** | **Grade 3** | **Any grade** | **Grade 1** | **Grade 2** | **Grade 3** | **Any grade** |
| Blood and lymphatic system disorders | Anemia, n (%) | 2 (11.1) | 0 | 0 | 2 (11.1) | 3 (23.1) | 1 (7.7) | 0 | 4 (30.8) |
| Cardiac disorders | Left ventricular systolic dysfunction, n (%) | 0 | 1 (5.6) | 0 | 1 (5.6) | 0 | 0 | 0 | 0 |
| Gastrointestinal disorders | Constipation, n (%) | 2 (11.1) | 0 | 0 | 2 (11.1) | 0 | 0 | 0 | 0 |
|  | Diarrhea, n (%) | 0 | 0 | 0 | 0 | 1 (7.7) | 0 | 0 | 1 (7.7) |
|  | Dry mouth, n (%) | 1 (5.6) | 0 | 0 | 1 (5.6) | 0 | 0 | 0 | 0 |
|  | Gastrointestinal pain, n (%) | 0 | 0 | 0 | 0 | 1 (7.7) | 0 | 0 | 1 (7.7) |
|  | Nausea, n (%) | 0 | 0 | 0 | 0 | 1 (7.7) | 0 | 0 | 1 (7.7) |
|  | Oral dysesthesia, n (%) | 0 | 0 | 0 | 0 | 1 (7.7) | 0 | 0 | 1 (7.7) |
| General disorders and administration site conditions | Chills, n (%) | 1 (5.6) | 0 | 0 | 1 (5.6) | 2 (15.4) | 0 | 0 | 2 (15.4) |
|  | Edema limbs, n (%) | 0 | 0 | 0 | 0 | 1 (7.7) | 0 | 0 | 1 (7.7) |
|  | Fatigue, n (%) | 0 | 0 | 0 | 0 | 4 (30.8) | 0 | 0 | 4 (30.8) |
|  | Fever, n (%) | 1 (5.6) | 0 | 0 | 1 (5.6) | 2 (15.4) | 0 | 0 | 2 (15.4) |
|  | Flu like symptoms, n (%) | 0 | 0 | 0 | 0 | 2 (15.4) | 0 | 0 | 2 (15.4) |
|  | General disorders and administration site conditions - Other, night sweats, n (%) | 0 | 0 | 0 | 0 | 1 (7.7) | 0 | 0 | 1 (7.7) |
|  | Non-cardiac chest pain, n (%) | 0 | 0 | 0 | 0 | 1 (7.7) | 0 | 0 | 1 (7.7) |
| Infections and infestations | Catheter related infection, n (%) | 1 (5.6) | 0 | 0 | 1 (5.6) | 0 | 0 | 0 | 0 |
| Investigations | Alanine aminotransferase increased, n (%) | 0 | 0 | 0 | 0 | 1 (7.7) | 0 | 0 | 1 (7.7) |
|  | Blood bilirubin increased, n (%) | 0 | 0 | 0 | 0 | 2 (15.4) | 0 | 0 | 2 (15.4) |
|  | CPK increased, n (%) | 0 | 0 | 0 | 0 | 1 (7.7) | 0 | 0 | 1 (7.7) |
|  | Creatinine increased, n (%) | 1 (5.6) | 0 | 0 | 1 (5.6) | 0 | 0 | 0 | 0 |
|  | Electrocardiogram QT corrected interval prolonged, n (%) | 1 (5.6) | 0 | 0 | 1 (5.6) | 0 | 0 | 0 | 0 |
|  | INR increased, n (%) | 0 | 0 | 0 | 0 | 1 (7.7) | 0 | 0 | 1 (7.7) |
|  | Investigations - Other, n (%) | 1 (5.6) | 0 | 0 | 1 (5.6) | 2 (15.4) | 0 | 0 | 2 (15.4) |
|  | Lipase increased, n (%) | 0 | 1 (5.6) | 0 | 1 (5.6) | 0 | 0 | 0 | 0 |
|  | Lymphocyte count decreased, n (%) | 0 | 0 | 0 | 0 | 3 (23.1) | 1 (7.7) | 0 | 4 (30.8) |
|  | Neutrophil count decreased, n (%) | 1 (5.6) | 0 | 1 (5.6) | 2 (11.1) | 2 (15.4) | 3 (23.1) | 1 (7.7) | 6 (46.2) |
|  | Platelet count decreased, n (%) | 2 (11.1) | 0 | 0 | 2 (11.1) | 0 | 0 | 0 | 0 |
|  | Serum amylase increased, n (%) | 0 | 1 (5.6) | 0 | 1 (5.6) | 0 | 1 (7.7) | 0 | 1 (7.7) |
|  | White blood cell decreased, n (%) | 0 | 0 | 0 | 0 | 3 (23.1) | 3 (23.1) | 0 | 6 (46.2) |
| Metabolism and nutrition disorders | Hyperkalemia, n (%) | 0 | 0 | 0 | 0 | 1 (7.7) | 0 | 0 | 1 (7.7) |
|  | Hyperuricemia, n (%) | 1 (5.6) | 0 | 0 | 1 (5.6) | 1 (7.7) | 0 | 0 | 1 (7.7) |
|  | Hypokalemia, n (%) | 1 (5.6) | 1 (5.6) | 0 | 2 (11.1) | 0 | 0 | 0 | 0 |
|  | Hyponatremia, n (%) | 0 | 0 | 0 | 0 | 1 (7.7) | 0 | 0 | 1 (7.7) |
| Musculoskeletal and connective tissue disorders | Back pain, n (%) | 0 | 0 | 0 | 0 | 1 (7.7) | 0 | 1 (7.7) | 2 (15.4) |
|  | Musculoskeletal and connective tissue disorder - Other, n (%) | 1 (5.6) | 0 | 0 | 1 (5.6) | 0 | 0 | 0 | 0 |
|  | Pain in extremity, n (%) | 0 | 0 | 0 | 0 | 3 (23.1) | 1 (7.7) | 0 | 4 (30.8) |
| Nervous system disorders | Dizziness, n (%) | 0 | 0 | 0 | 0 | 1 (7.7) | 1 (7.7) | 0 | 2 (15.4) |
|  | Dysesthesia, n (%) | 0 | 0 | 0 | 0 | 1 (7.7) | 0 | 0 | 1 (7.7) |
|  | Headache, n (%) | 0 | 0 | 0 | 0 | 1 (7.7) | 0 | 0 | 1 (7.7) |
|  | Paresthesia, n (%) | 1 (5.6) | 0 | 0 | 1 (5.6) | 1 (7.7) | 0 | 0 | 1 (7.7) |
|  | Peripheral sensory neuropathy, n (%) | 0 | 0 | 0 | 0 | 1 (7.7) | 0 | 0 | 1 (7.7) |
| Psychiatric disorders | Anxiety, n (%) | 1 (5.6) | 0 | 0 | 1 (5.6) | 0 | 0 | 0 | 0 |
| Respiratory, thoracic and mediastinal disorders | Cough, n (%) | 1 (5.6) | 0 | 0 | 1 (5.6) | 0 |  | 0 | 0 |
|  | Sore throat, n (%) | 0 | 0 | 0 | 0 | 1 (7.7) | 0 | 0 | 1 (7.7) |
| Skin and subcutaneous tissue disorders | Dry skin, n (%) | 0 | 0 | 0 | 0 | 1 (7.7) | 0 | 0 | 1 (7.7) |
| Vascular disorders | Hypertension, n (%) | 0 | 2 (11.1) | 0 | 2 (11.1) | 0 | 0 | 0 | 0 |
|  | Hypotension, n (%) | 1 (5.6) | 0 | 0 | 1 (5.6) | 1 (7.7) | 0 | 0 | 1 (7.7) |

Adverse events (AEs) and serious AEs are classified according to CTCAE V4.03. Severity and relationship were judged by the investigator. AEs are reported until the primary safety endpoint, i.e. until V7 or V9a for cohorts 1-5 or cohort 6, respectively. Patient population was grouped according to received dose of FLYSYN: cohorts 1-4 (15mg/m^2^) and cohorts 5-6 (>15mg/m^2^). For each patient the adverse event occurring at least once was counted with the highest CTCAE grading. For “Investigations – Other”, interleukin 2 receptor increased, ferritin increased and C-reactive protein increased were reported. For “Musculoskeletal and connective tissue disorder – Other” whole body pain and snapping finder (left thumb) were reported. CTCAE, Common Terminology Criteria for Adverse Events; n, number; SOC, system organ class; WBC, white blood cell

## **Supplementary Table S5:** Treatment-related adverse events by dose of FLYSYN

|  | | **Cohorts 1-4 (n=18)** | | | | **Cohorts 5-6 (n=13)** | | | |
| --- | --- | --- | --- | --- | --- | --- | --- | --- | --- |
| **System organ class** | **CTCAE term** | **Grade 1** | **Grade 2** | **Grade 3** | **Any grade** | **Grade 1** | **Grade 2** | **Grade 3** | **Any grade** |
| Blood and lymphatic system disorders | Anemia, n (%) | 2 (11.1) | 0 | 0 | 2 (11.1) | 3 (23.1) | 1 (7.7) | 0 | 4 (30.8) |
| Gastrointestinal disorders | Nausea, n (%) | 0 | 0 | 0 | 0 | 1 (7.7) | 0 | 0 | 1 (7.7) |
| General disorders and administration site conditions | Chills, n (%) | 1 (5.6) | 0 | 0 | 1 (5.6) | 1 (7.7) | 0 | 0 | 1 (7.7) |
|  | Edema limbs, n (%) | 0 | 0 | 0 | 0 | 1 (7.7) | 0 | 0 | 1 (7.7) |
|  | Fatigue, n (%) | 0 | 0 | 0 | 0 | 3 (23.1) | 0 | 0 | 3 (23.1) |
|  | Fever, n (%) | 1 (5.6) | 0 | 0 | 1 (5.6) | 2 (15.4) | 0 | 0 | 2 (15.4) |
|  | Flu like symptoms, n (%) | 0 | 0 | 0 | 0 | 2 (15.4) | 0 | 0 | 2 (15.4) |
|  | Non-cardiac chest pain, n (%) | 0 | 0 | 0 | 0 | 1 (7.7) | 0 | 0 | 1 (7.7) |
| Investigations | Blood bilirubin increased, n (%) | 0 | 0 | 0 | 0 | 2 (15.4) | 0 | 0 | 2 (15.4) |
|  | Investigations - Other, interleukine 2 receptor increased n (%) | 0 | 0 | 0 | 0 | 1 (7.7) | 0 | 0 | 1 (7.7) |
|  | Lymphocyte count decreased, n (%) | 0 | 0 | 0 | 0 | 3 (23.1) | 1 (7.7) | 0 | 4 (30.8) |
|  | Neutrophil count decreased, n (%) | 0 | 0 | 1 (5.6) | 1 (5.6) | 2 (15.4) | 3 (23.1) | 1 (7.7) | 6 (46.2) |
|  | Platelet count decreased, n (%) | 2 (11.1) | 0 | 0 | 2 (11.1) | 0 | 0 | 0 | 0 |
|  | WBC decreased, n (%) | 0 | 0 | 0 | 0 | 3 (23.1) | 3 (23.1) | 0 | 6 (46.2) |
| Musculoskeletal and connective tissue disorders | Back pain, n (%) | 0 | 0 | 0 | 0 | 0 | 0 | 1 (7.7) | 1 (7.7) |
|  | Pain in extremity, n (%) | 0 | 0 | 0 | 0 | 1 (7.7) | 1 (7.7) | 0 | 2 (15.4) |
| Nervous system disorders | Dizziness, n (%) | 0 | 0 | 0 | 0 | 1 (7.7) | 1 (7.7) | 0 | 2 (15.4) |
|  | Dysesthesia, n (%) | 0 | 0 | 0 | 0 | 1 (7.7) | 0 | 0 | 1 (7.7) |
|  | Headache, n (%) | 0 | 0 | 0 | 0 | 1 (7.7) | 0 | 0 | 1 (7.7) |
|  | Paresthesia, n (%) | 1 (5.6) | 0 | 0 | 1 (5.6) | 1 (7.7) | 0 | 0 | 1 (7.7) |
| Vascular disorders | Hypertension, n (%) | 0 | 1 (5.6) | 0 | 1 (5.6) | 0 | 0 | 0 | 0 |
|  | Hypotension, n (%) | 1 (5.6) | 0 | 0 | 1 (5.6) | 1 (7.7) | 0 | 0 | 1 (7.7) |

Adverse events (AEs) and serious AEs are classified according to CTCAE V4.03. Severity and relationship were judged by the investigator. AEs are reported until the primary safety endpoint, i.e. until V7 or V9a for cohorts 1-5 or cohort 6, respectively. Patient population was grouped according to received dose of FLYSYN: cohorts 1-4 (15mg/m^2^) and cohorts 5-6 (>15mg/m^2^). For each patient the adverse event occurring at least once was counted with the highest CTCAE grading. CTCAE, Common Terminology Criteria for Adverse Events; n, number; SOC, system organ class; WBC, white blood cell.

## **Supplementary Table S6:** Treatment-emergent adverse events all cohorts until visit 11

|  | | **All subjects (n=31)** | | | |
| --- | --- | --- | --- | --- | --- |
| **System organ class** | **CTCAE term** | **Grade 1** | **Grade 2** | **Grade 3** | **Any grade** |
| Patients with events | All terms, n (%) | 12 (38.7) | 11 (35.5) | 3 (9.7) | 26 (84) |
| Blood and lymphatic system disorders | Anemia, n (%) | 5 (16.1) | 1 (3.2) | 0 | 6 (19.4) |
| Cardiac disorders | Left ventricular systolic dysfunction, n (%) | 0 | 1 (3.2) | 0 | 1 (3.2) |
| Gastrointestinal disorders | Constipation, n (%) | 2 (6.5) | 0 | 0 | 2 (6.5) |
|  | Diarrhea, n (%) | 1 (3.2) | 0 | 0 | 1 (3.2) |
|  | Dry mouth, n (%) | 1 (3.2) | 0 | 0 | 1 (3.2) |
|  | Gastrointestinal disorders – Other, coated tongue, n (%) | 1 (3.2) | 0 | 0 | 1 (3.2) |
|  | Gastrointestinal pain, n (%) | 1 (3.2) | 0 | 0 | 1 (3.2) |
|  | Nausea, n (%) | 1 (3.2) | 0 | 0 | 1 (3.2) |
|  | Oral dysesthesia, n (%) | 1 (3.2) | 0 | 0 | 1 (3.2) |
| General disorders and administration site conditions | Chills, n (%) | 3 (9.7) | 0 | 0 | 3 (9.7) |
|  | Edema limbs, n (%) | 1 (3.2) | 0 | 0 | 1 (3.2) |
|  | Fatigue, n (%) | 4 (12.9) | 0 | 0 | 4 (12.9) |
|  | Fever, n (%) | 3 (9.7) | 0 | 0 | 3 (9.7) |
|  | Flu like symptoms, n (%) | 3 (9.7) | 0 | 0 | 3 (9.7) |
|  | General disorders and administration site conditions – Other, night sweats, n (%) | 1 (3.2) | 0 | 0 | 1 (3.2) |
|  | Non-cardiac chest pain, n (%) | 1 (3.2) | 0 | 0 | 1 (3.2) |
| Infections and infestations | Catheter related infection, n (%) | 1 (3.2) | 0 | 0 | 1 (3.2) |
|  | Pharyngitis, n (%) | 1 (3.2) | 0 | 0 | 1 (3.2) |
|  | Upper respiratory infection, n (%) | 0 | 1 (3.2) | 0 | 1 (3.2) |
|  | Urinary tract infection, n (%) | 0 | 2 (6.5) | 0 | 2 (6.5) |
| Investigations | Alanine aminotransferase increased, n (%) | 1 (3.2) | 0 | 0 | 1 (3.2) |
|  | Blood bilirubin increased, n (%) | 2 (6.5) | 0 | 0 | 2 (6.5) |
|  | CPK increased, n (%) | 2 (6.5) | 1 (3.2) | 0 | 3 (9.7) |
|  | Creatinine increased, n (%) | 1 (3.2) | 0 | 0 | 1 (3.2) |
|  | Electrocardiogram QT corrected interval prolonged, n (%) | 1 (3.2) | 0 | 0 | 1 (3.2) |
|  | INR increased, n (%) | 1 (3.2) | 0 | 0 | 1 (3.2) |
|  | Investigations - Other, n (%) | 3 (9.7) | 0 | 0 | 3 (9.7) |
|  | Lipase increased, n (%) | 0 | 1 (3.2) | 0 | 1 (3.2) |
|  | Lymphocyte count decreased, n (%) | 3 (9.7) | 1 (3.2) | 0 | 4 (12.9) |
|  | Neutrophil count decreased, n (%) | 3 (9.7) | 3 (9.7) | 2 (6.5) | 8 (25.8) |
|  | Platelet count decreased, n (%) | 2 (6.5) | 0 | 0 | 2 (6.5) |
|  | Serum amylase increased, n (%) | 0 | 2 (6.5) | 0 | 2 (6.5) |
|  | White blood cell decreased, n (%) | 3 (9.7) | 3 (9.7) | 0 | 6 (19.4) |
| Metabolism and nutrition disorders | Hyperkalemia, n (%) | 1 (3.2) | 0 | 0 | 1 (3.2) |
|  | Hyperuricemia, n (%) | 2 (6.5) | 0 | 0 | 2 (6.5) |
|  | Hypokalemia, n (%) | 1 (3.2) | 1 (3.2) | 0 | 2 (6.5) |
|  | Hyponatremia, n (%) | 1 (3.2) | 0 | 0 | 1 (3.2) |
| Musculoskeletal and connective tissue disorders | Arthralgia, n (%) | 1 (3.2) | 0 | 0 | 1 (3.2) |
|  | Back pain, n (%) | 1 (3.2) | 0 | 1 (3.2) | 2 (6.5) |
|  | Musculoskeletal and connective tissue disorder - Other, n (%) | 2 (6.5) | 0 | 0 | 2 (6.5) |
|  | Pain in extremity, n (%) | 4 (12.9) | 1 (3.2) | 0 | 5 (16.1) |
| Nervous system disorders | Dizziness, n (%) | 1 (3.2) | 1 (3.2) | 0 | 2 (6.5) |
|  | Dysesthesia, n (%) | 1 (3.2) | 0 | 0 | 1 (3.2) |
|  | Headache, n (%) | 1 (3.2) | 0 | 0 | 1 (3.2) |
|  | Paresthesia, n (%) | 2 (6.5) | 0 | 0 | 2 (6.5) |
|  | Peripheral sensory neuropathy, n (%) | 1 (3.2) | 0 | 0 | 1 (3.2) |
| Psychiatric disorders | Anxiety, n (%) | 1 (3.2) | 0 | 0 | 1 (3.2) |
| Respiratory, thoracic and mediastinal disorders | Cough, n (%) | 1 (3.2) | 0 | 0 | 1 (3.2) |
|  | Sore throat, n (%) | 1 (3.2) | 0 | 0 | 1 (3.2) |
| Skin and subcutaneous tissue disorders | Dry skin, n (%) | 1 (3.2) | 0 | 0 | 1 (3.2) |
| Vascular disorders | Hypertension, n (%) | 0 | 2 (6.5) | 0 | 2 (6.5) |
|  | Hypotension, n (%) | 2 (6.5) | 0 | 0 | 2 (6.5) |

Adverse events (AEs) and serious AEs are classified according to CTCAE V4.03. Severity and relationship were judged by the investigator. AEs are reported until the secondary safety endpoint, i.e. until 180 days after first study drug application. For each patient the adverse event occurring at least once was counted with the highest CTCAE grading. For “Investigations – Other”, interleukin 2 receptor increased, ferritin increased and C-reactive protein increased were reported. For “Musculoskeletal and connective tissue disorder – Other” whole body pain and snapping finder (left thumb) were reported. CTCAE, Common Terminology Criteria for Adverse Events; n, number; SOC, system organ class; WBC, white blood cell.

## **Supplementary Table S7**: Reasons for study follow-up discontinuation prior to EOS visit

| **Reasons** | | **N (%)** | |
| --- | --- | --- | --- |
|  | Discontinuation | 25 (80.7) |  |
|  | Allo-HCT | 6 (19.4) |  |
|  | Progressive disease | 17 (54.8) |  |
|  | Alternative treatment | 1 (3.2) |  |
|  | Death | 0 |  |
|  | AEs related to study drug | 0 |  |
|  | Lost to follow up | 1 (3.2) |  |

Progressive disease is defined as either overt hematologic relapse or MRD progression (≥1 log increase compared to MRD level prior to FLYSYN). Allo-HCT, allogeneic hematopoietic-cell transplantation

## **Supplementary Table S8:** Pharmacokinetics of FLYSYN by cohort

|  | **Cohort 1-6** | **Cohort 2** | **Cohort 3** | **Cohort 4** | **Cohort 5** | **Cohort 6** | **Cohort 3-6** |
| --- | --- | --- | --- | --- | --- | --- | --- |
|  | **0.5 mg/m^2^**  **(n = 31)** | **1.5 mg/m^2^**  **(n = 3)** | **5 mg/m^2^**  **(n = 3)** | **15 mg/m^2^**  **(n = 9)** | **45 mg/m^2^**  **(n = 3)** | **3 x 15 mg/m^2^ combined**  **(n = 7)** | **(n=22)** |
| T_max_, in h, median (range) | 3.0  (1.5 – 18.0) | 6.0  (3.0 – 24.0) | 3.0  (3.0 – 6.0) | 6.0  (3.0 – 18.0) | 20.5  (18.0 – 22.0) | 649.0  (318.0 – 697.7) | 12.0  (3.0 – 697.7) |
| C_max_, in ng/ml, median (range) | 253  (44 – 7,135) | 510  (435 – 1,719) | 3,018  (2,636 – 5,337) | 7,431  (479 – 13,030) | 23,100  (21,444 – 24,301) | 16,794  (11,079 – 19,549) | 11,207  (2,636 – 24,301) |
| AUC_0-t_, median (range) | 3,060  (46 – 44,916) | 70,022  (59,457 – 323,055) | 458,128  (450,279 – 683,278) | 1,386,771  (580,154 – 20,411,435) | 7,099,111  (1,751,814 – 7,495,924) | 7,462,091  (3,434,363 – 9,091,749) | 2,816,737  (450,279 – 20,411,435) |
| AUC_0-∞_, median (range) | - | - | 487,067  (456,861 – 722,756) | 1,461,852  (584,096 – 3,246,818) | 7,116,966  (1,765,730 – 7,694,991) | 8,104,464  (3,444,000 – 9,937,290) | 2,608,426  (456,861 – 9,937,290) |
| AUC_0-∞_ /dose, median (range) | - | - | 108,237  (101,524 – 160,612) | 100,817  (40,283 – 223,919) | 159,932  (39,679 – 172,921) | 182,123  (77,394 – 223,310) | 109,236  (39,679 – 172,921) |
| T_1/2_, in h, median (range) | - | - | 117.99  (110.44 – 193.99) | 178.99  (45.29 – 1849.81) | 188.04  (70.84 – 255.23) | 166.27  (87.37 – 449.58) | 172.63  (45.29 – 1849.81) |
| K_el_, in 1/h, median (range) | - | - | 0.0059  (0.0036 – 0.0063) | 0.0039  (0.0004 – 0.0153) | 0.0037  (0.0031 – 0.0098) | 0.0042  (0.0015 – 0.0079) | 0.0041  (0.0004 – 0.0153) |

Pharmacokinetics of FLYSYN are displayed for each cohort. Parameters were assessed after start of infusion on day 1 and day 2 for cohorts 1 and 2-6, respectively. In cohorts 4 and 6 one and two patients, respectively, were not assessable for pharmacokinetics. AUC, area under the curve; c, concentration, T, time; T_1/2_, half- life.

## **Supplementary Table S9:** Response to FLYSYN treatment

| **Response to FLYSYN** | **All**  **(n = 31)** | **Cohorts 1-4**  **(n = 18)** | **Cohorts 5-6**  **(n = 13)** |
| --- | --- | --- | --- |
| MRD reduction rate^₮^, n (%) | 20 (65) | 10 (56) | 10 (77) |
| MRD response rate^¥^, n (%) | 11 (35) | 5 (28) | 6 (46) |
| MRD negativity rate^⁑^, n (%) | 6 (19) | 4 (22) | 2 (15) |
| Baseline^†^ adjusted MRD response rate^¥^ in MRD_low_ patients, n (%) | 6 (60) | 3 (60) | 3 (60) |
| Baseline^†^ adjusted MRD response rate^¥^ in MRD_high_ patients, n (%) | 4 (22) | 1 (9) | 3 (43) |
| Time to best MRD response in days, median (range) | 29 (15-92) | 29 (15-43) | 50.5 (15-92) |
| MRD response rate at visit 9, n (%) | 3 (14) | 2 (17) | 1 (10) |
| Missing data at visit 9, n (%) | 9 (29) | 6 (33) | 3 (23) |

MRD assessment was routinely performed during follow-up. ^₮^MRD reduction is defined as any reduction from baseline until Visit 10 after treatment; ^¥^MRD response is defined as a log reduction from baseline or MRD negativity until Visit 10 after treatment; ^⁑^ baseline NPM1 levels in these patients were in 11 (median, range 5-103) NPM1/10,000 ABL copies; ^†^ only patients monitored for NPM1 were analyzed; 200 NPM1/10,000 ABL copies was chosen as cut-off to divide patients at baseline into MRD_low_ (≤200 NPM1/10,000 ABL copies) and MRD_high_ (>200 NPM1/10,000 ABL copies); MRD, measurable residual disease; n, number.

## **Supplementary Table S10:** Evidence of progressive disease (EPD) free survival after FLYSYN treatment

|  | **All**  **(n = 31)** | **Cohorts 1-4**  **(n = 18)** | **Cohorts 5-6**  **(n = 13)** |
| --- | --- | --- | --- |
| Patients with event, n (%) | 17 (55%) | 10 (55%) | 7 (54%) |
| Median, months | 6.9 | 11.7 | 6.9 |
| 95% CI, months | (3, not reached) | (3, 13.4) | (1.4, not reached) |

Time to evidence of progressive disease (EPD) was calculated from first dosing of FLYSYN until disease progression defined as either hematologic or molecular relapse. The latter was defined as one log increase in MRD level compared to MRD level prior to first application of FLYSYN. 95% CIs for survival probabilities and median survival were calculated based on a log-log transform of the survival function estimate; CI, confidence interval.

## **Supplementary Table S11:** Development of anti-drug antibodies (ADAs) by cohort

|  | | **Cohort 1** | **Cohort 2** | **Cohort 3** | **Cohort 4** | **Cohort 5** | **Cohort 6** |
| --- | --- | --- | --- | --- | --- | --- | --- |
|  | | **0.5 mg/m^2^ (n = 3)** | **1.5 mg/m^2^ (n = 3)** | **5 mg/m^2^ (n = 3)** | **15 mg/m^2^ (n = 9)** | **45 mg/m^2^ (n = 3)** | **3 x 15 mg/m^2^ (n = 10)** |
|  | Positive anti-drug antibodies | 0 | 0 | 0 | 0 | 0 | 0 |

## **Supplementary Table S12:** List of prohibited medications within 14 days prior to the first dose of study drug

| **Immunosuppresive drugs** | Kinase Inhibitos | Cytotoxic agents |
| --- | --- | --- |
| Glucocorticoids (Immunosuppressive mechanism, anti-inflammatory effects | Alectinib (Alecensa®), Axitinib (Inlyta®), Cabozantinib (Cometriq®), Crizotinib (Xalkori®), Lapatinib (Tyverb®), Lenvatinib (Lenvima®), Pazopanib (Votrient®), Regorafenib (Stivarga®), Sunitinib (Sutent®), Sorafenib (Nexavar®), Vandetanib (Caprelsa®) | Actinomycin, All-trans retinoic acid, Azacitidine, Azathioprine, Bleomycin, Bortezomib, Carboplatin, Capecitabine, Cisplatin, Chlorambucil, Cyclophosphamide, Cytarabine, Daunorubicin, Docetaxel, Doxifluridine, Doxorubicin, Epirubicin, Epothilone, Etoposide, Fluorouracil, Gemcitabine, Hydroxyurea, Idarubicin, Imatinib, Irinotecan, Mechlorethamine, Mercaptopurine, Methotrexate, Mitoxantrone, Oxaliplatin, Paclitaxel, Pemetrexed, Teniposide, Tioguanine, Topotecan, Valrubicin, Vinblastine, Vincristine, Vindesine, Vinorelbine |
| Cytostatics (alkylating agents, antimetabolites) | Epidermal Growth Factor Receptor Tyrokinase Inhibitors (EGFR-TKIs): Afatinib (Gilotrif®), Erlotinib (Tarceva®), Gefitinib (Iressa®), Osimertinib, (Tagrisso®) |  |
| Antibodies (polyclonal antibodies, monoclonal antibodies) | Breakpoint Cluster Region - Abelson Murine Leukemia – Inhibitors (BCR-ABL): Bosutinib (Bosulif®), Imatinib (Glivec®), Nilotinib (Tasigna®), Dasatinib (Sprycel®)  Januskinase-Inhibitors:, Ruxolitinib (Jakafi®), Tofacitinib (Xeljanz®) |  |
| Drugs acting on immunophilins (Ciclosporin, Tacrolimus, Sirolimus) | Mechanistic Target of Rapamycin-Inhibitors (mTOR) : Everolimus (Afinitor®), Sirolimus (= Rapamycin, Rapamune®), Temsirolimus (Torisel®) |  |
| Other drugs (Interferons, Opioids, TNF binding proteins, Mycophenolate, small biological agents) | B-Rapidly Accelerated Fibrosarcoma-Inhibitors (BRAF): Vemurafenib (Zelboraf®), Dabrafenib (Tafinlar®)  Mitogen Actvated Protein Kinase-Inhibitors (MEK): Trametinib (Mekinist®) |  |
|  | Bruton's Tyrosine Kinase-Inhibitors (BTK): Ibrutinib (Imbruvica®) |  |
|  | Cyclin-dependent Kinase: Palbociclib (Ibrance®) |  |

## **Supplementary Table S13:** Detailed information on patients’ prior therapies and remission status

| Patients‘ treatment | **Median number of cycles (range)** |
| --- | --- |
| 3 + 7 induction cycles | 2 (1 - 2) |
| 3 + 7 consolidation cycles | 3 (2 - 4) |
| 3 + 7 + midostaurin induction cycles | 2 (1 - 2) |
| 3 + 7 + midostaurin consolidation cycles | 3 (3) |
| A-ICE induction cycles | 2 (2) |
| A-ICE consolidation cycles | 3 (3) |
| Cytarabine etoposide | 6 (6) |
| Decitabine | 14 (14) |
| GO-A-ICE induction cycles | 2 (2) |
| GO-A-ICE consolidation cycles | 3 (3) |
| GO-ICE induction cycles | 2 (2) |
| GO-ICE consolidation cycles | 3 (3) |
| ICE induction cycles | 2 (2) |
| ICE consolidation cycles | 3 (3) |
| Patients with first CR after therapy | 30 (97%) |

A-ICE, all-trans retoinic acid, idarubicine, cytarabine, etoposide; GO, gemtuzumab ozogamicin

# Supplementary Figures

**
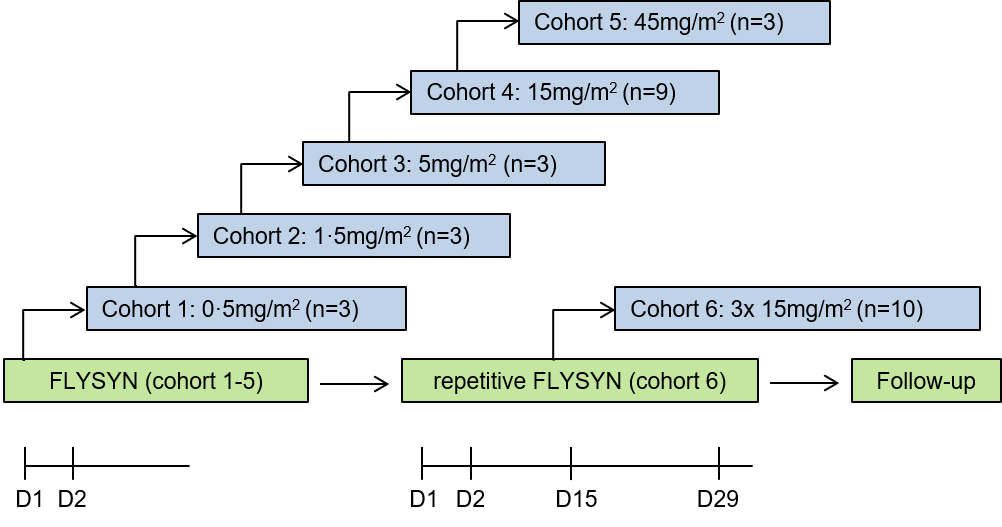
Supplementary Figure S1: Overview of trial design and dosing.** Patients were treated according to a rule-based 3+3 design. A cohort was determined as safe if in 0 of 3 patients no dose limiting toxicity was observed. Cohorts 4 and 6 could be expanded to 9 and 10 patients, respectively. Cohorts 2, 3, 4 and 5 applied 0.5 mg/m^2^ body surface area (BSA) on day 1 prior to escalating to a total dose of 1.5 mg/m^2^ BSA, 5 mg/m^2^ BSA, 15 mg/m^2^ BSA, 45 mg/m^2^ BSA on the consecutive day, respectively. In cohort 6, patients received 15 mg/m^2^ BSA on days 2, 15 and 29 with a test dose on day 1 (0.5 mg/m^2^ BSA). After each cohort a DSMB decision was required prior to dosing with the next higher dose level.

**
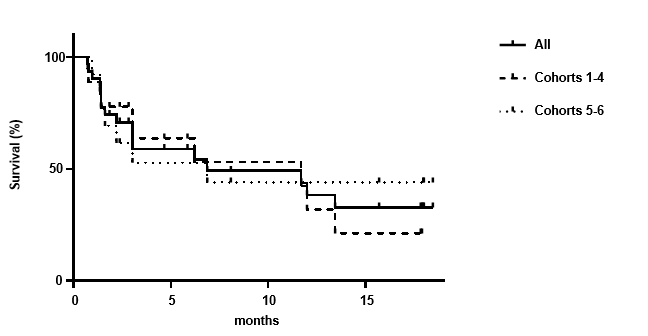
**

**Supplementary Figure S2:** **Evidence of progressive disease (EPD) free survival.** Time to evidence of progressive disease (EPD) was calculated from first dosing of FLYSYN until disease progression defined as either hematologic or molecular relapse. Kaplan-Meier analysis of all patients treated with FLYSYN (continuous line), cohorts 1-4 (dashed line) and cohorts 5-6 (dotted line). Median of time to EPD free survival in all patients was 6.9 months.

**
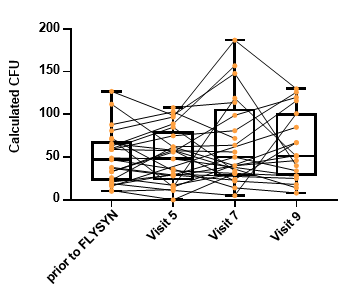
**

**Supplementary Figure S3: Assessment of colony forming units during FLYSYN treatment.** Before and during FLYSYN treatment bone marrow samples were obtained to perform colony forming unit (CFU) assays. Dots display individual patients. Combined box-line plots display median with 25^th^ or 75^th^ percentiles, and min/max whiskers.

**
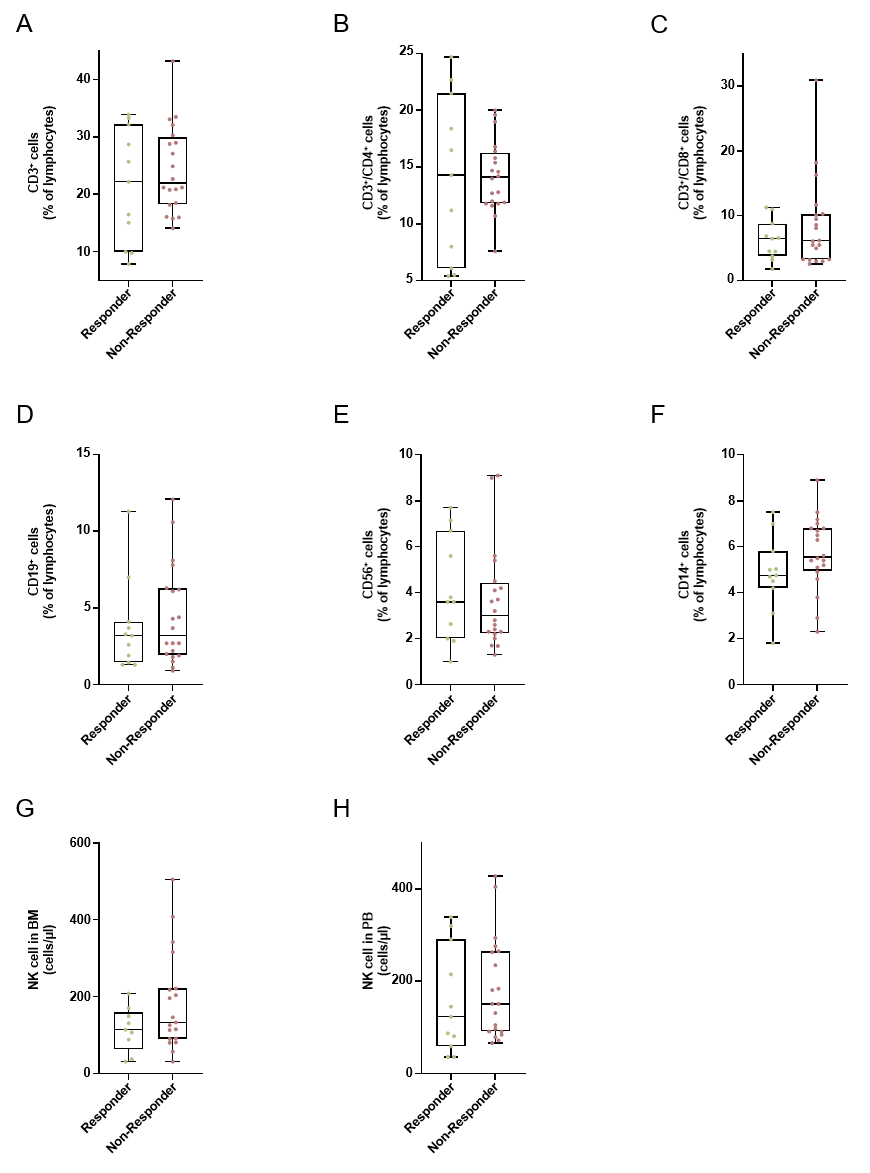
**

**Supplementary Figure S4: Lymphocyte subsets according to response to FLYSYN treatment.** Lymphocyte subsets were determined prior to application of FLYSYN. Percentage of lymphocyte subsets are displayed for (A) CD3^+^, (B) CD3^+^CD4^+^, (C) CD3^+^CD8^+^, (D) CD19^+^, (E) CD56^+^ and (F) CD14^+^ as determined by immunophenotyping prior to FLYSYN application. Absolute number of NK cells are displayed for (G) bone marrow and (H) peripheral blood. Green dots indicate trial subjects responding to FLYSYN treatment (Responder), i.e. MRD reduction by >1 log or negativity in BM, whereas brown dots indicate trial subjects without MRD response. BM, bone marrow; PB, peripheral blood. Box plots show median with 25^th^ or 75^th^ percentiles, and min/max whiskers.

**
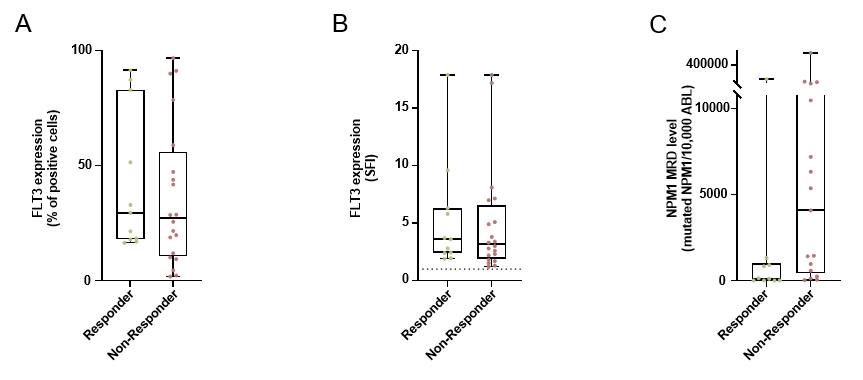
**

**Supplementary Figure S5: FLT3 expression and MRD level according to response to FLYSYN treatment.** (A) Percentage of FLT3 expression and (B) specific fluorescence intensity (SFI) were determined on residual blasts by flow cytometry prior to FLYSYN treatment. (C) Minimal residual disease (MRD) level in bone marrow were determined for NPM1 by quantitative polymerase chain reaction. MRD, minimal residual disease. Box plots show median with 25^th^ or 75^th^ percentiles, and min/max whiskers.

| **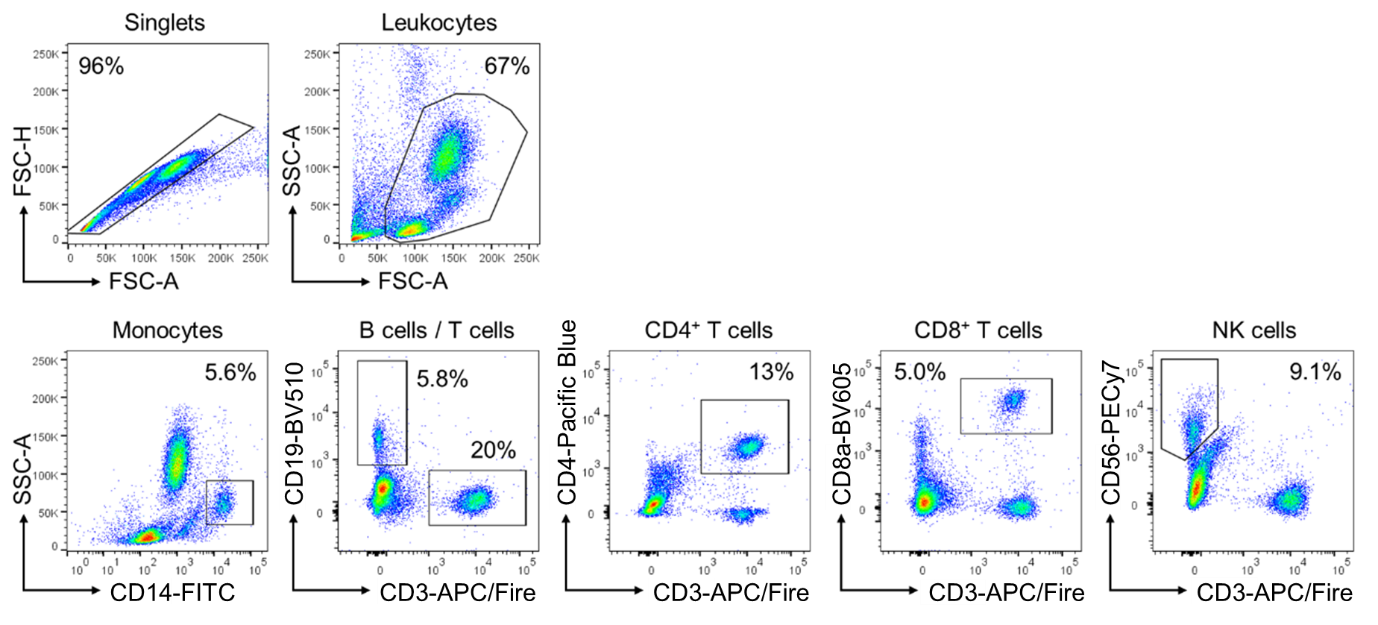** |
| --- |

**Supplementary Figure S6: Gating strategy for flow cytometry-based evaluation of immune cells and activation in whole blood samples**. Representative example showing the flow cytometry gating strategy for different immune cell populations. Hierarchical gating strategy was used to define single cells (FSC-A vs. FSC-H) and leucocytes (FSC-A vs. SSC-A). Within the leukocyte population, it was gated on monocytes (SCC-A vs. CD14), B and T cells (CD19 vs. CD3), CD4+ T cells (CD4 vs. CD3), CD8+ T cells (CD8 vs. CD3) and NK cells (CD56 vs. CD3).

**
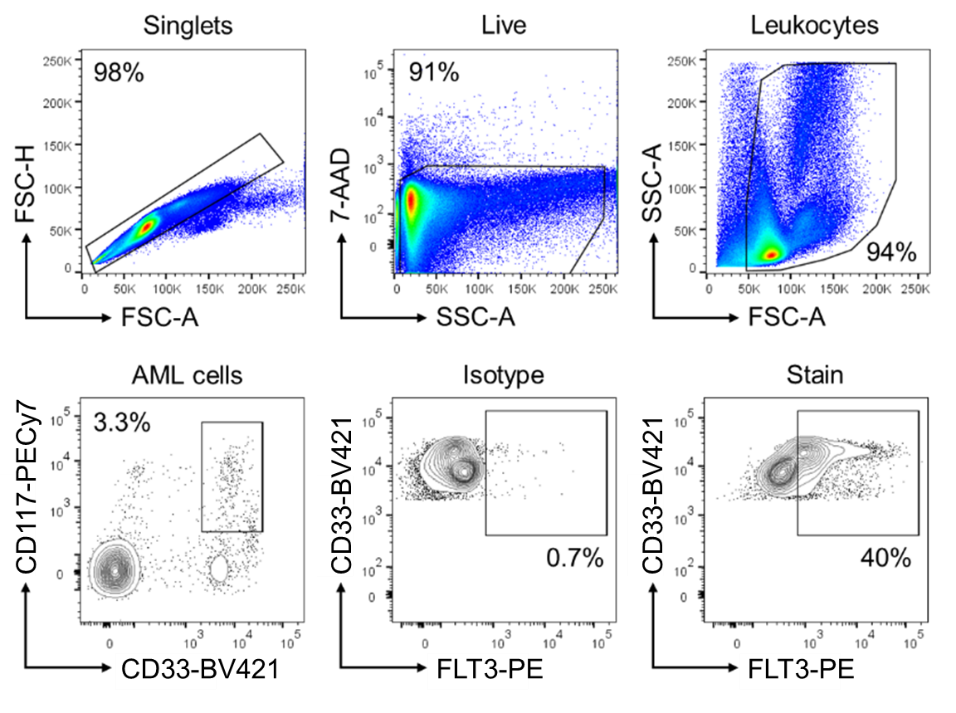
**

**Supplementary Figure S7: Gating strategy for flow cytometry-based analysis of FLT3 expression on AML cells in the bone marrow.** Representative example showing the flow cytometry gating strategy for AML cells in the bone marrow and FLT3 expression. Hierarchical gating strategy was used to define single cells (FSC-A vs. FSC-H), living cells (SSC-A vs. 7-AAD) and leucocytes (FSC-A vs. SSC-A). Within the leukocyte population, it was gated on AML cells according to patients’ specific immune phenotyping (CD33 vs. CD117). FLT3 expression was assessed using corresponding isotype controls (FLT3 vs. CD33).

**References**

1. Arber, D.A.*, et al.* The 2016 revision to the World Health Organization classification of myeloid neoplasms and acute leukemia. *Blood* **127**, 2391-2405 (2016).

2. Dohner, H.*, et al.* Diagnosis and management of AML in adults: 2022 recommendations from an international expert panel on behalf of the ELN. *Blood* **140**, 1345-1377 (2022).

3. Dohner, H.*, et al.* Diagnosis and management of AML in adults: 2017 ELN recommendations from an international expert panel. *Blood* **129**, 424-447 (2017).

4. Thol, F.*, et al.* Measurable residual disease monitoring by NGS before allogeneic hematopoietic cell transplantation in AML. *Blood* **132**, 1703-1713 (2018).

5. Kronke, J.*, et al.* Monitoring of minimal residual disease in NPM1-mutated acute myeloid leukemia: a study from the German-Austrian acute myeloid leukemia study group. *J Clin Oncol* **29**, 2709-2716 (2011).

6. Hofmann, M.*, et al.* Generation, selection and preclinical characterization of an Fc-optimized FLT3 antibody for the treatment of myeloid leukemia. *Leukemia* **26**, 1228-1237 (2012).
